# Somatic inactivating *PTPRJ* mutations and dysregulated pathways identified in canine malignant melanoma by integrated comparative genomic analysis

**DOI:** 10.1371/journal.pgen.1007589

**Published:** 2018-09-06

**Authors:** William P. D. Hendricks, Victoria Zismann, Karthigayini Sivaprakasam, Christophe Legendre, Kelsey Poorman, Waibhav Tembe, Nieves Perdigones, Jeffrey Kiefer, Winnie Liang, Valerie DeLuca, Mitchell Stark, Alison Ruhe, Roe Froman, Nicholas S. Duesbery, Megan Washington, Jessica Aldrich, Mark W. Neff, Matthew J. Huentelman, Nicholas Hayward, Kevin Brown, Douglas Thamm, Gerald Post, Chand Khanna, Barbara Davis, Matthew Breen, Alexander Sekulic, Jeffrey M. Trent

**Affiliations:** 1 Integrated Cancer Genomics Division, Translational Genomics Research Institute (TGen), Phoenix, Arizona, United States of America; 2 Department of Biomedical Informatics, Arizona State University, Phoenix, Arizona, United States of America; 3 Department of Molecular Biomedical Sciences, College of Veterinary Medicine, North Carolina State University, Raleigh, NC, United States of America; 4 Department of Dermatology, Mayo Clinic, Scottsdale, Arizona, United States of America; 5 School of Life Sciences, Arizona State University, Phoenix, Arizona, United States of America; 6 Dermatology Research Centre, The University of Queensland, The University of Queensland Diamantina Institute, Translational Research Institute, Woolloongabba, Queensland, Australia; 7 Veterinary Genetics Laboratory, University of California Davis, Davis, California, United States of America; 8 Laboratory of Cancer and Developmental Cell Biology, Van Andel Research Institute (VARI), Grand Rapids, Michigan, United States of America; 9 Spectrum Health, Grand Rapids, Michigan, United States of America; 10 Program in Canine Genetics and Genomics, Van Andel Research Institute (VARI), Grand Rapids, Michigan, United States of America; 11 Neurogenomics Division, Translational Genomics Research Institute (TGen), Phoenix, Arizona, United States of America; 12 Oncogenomics Laboratory, QIMR Berghofer Medical Research Institute, Herston, Brisbane, Queensland, Australia; 13 Division of Cancer Epidemiology and Genetics, National Cancer Institute, National Institutes of Health, Bethesda, Maryland, United States of America; 14 Flint Animal Cancer Center, Colorado State University, Fort Collins, Colorado, United States of America; 15 The Veterinary Cancer Center, Norwalk, Connecticut, United States of America; 16 Innogenics Inc., Harvard, Massachusetts, United States of America; 17 Comparative Medicine Institute, North Carolina State University, Raleigh, NC, United States of America; HudsonAlpha Institute for Biotechnology, UNITED STATES

## Abstract

Canine malignant melanoma, a significant cause of mortality in domestic dogs, is a powerful comparative model for human melanoma, but little is known about its genetic etiology. We mapped the genomic landscape of canine melanoma through multi-platform analysis of 37 tumors (31 mucosal, 3 acral, 2 cutaneous, and 1 uveal) and 17 matching constitutional samples including long- and short-insert whole genome sequencing, RNA sequencing, array comparative genomic hybridization, single nucleotide polymorphism array, and targeted Sanger sequencing analyses. We identified novel predominantly truncating mutations in the putative tumor suppressor gene *PTPRJ* in 19% of cases. No *BRAF* mutations were detected, but activating *RAS* mutations (24% of cases) occurred in conserved hotspots in all cutaneous and acral and 13% of mucosal subtypes. *MDM2* amplifications (24%) and *TP53* mutations (19%) were mutually exclusive. Additional low-frequency recurrent alterations were observed amidst low point mutation rates, an absence of ultraviolet light mutational signatures, and an abundance of copy number and structural alterations. Mutations that modulate cell proliferation and cell cycle control were common and highlight therapeutic axes such as MEK and MDM2 inhibition. This mutational landscape resembles that seen in *BRAF* wild-type and sun-shielded human melanoma subtypes. Overall, these data inform biological comparisons between canine and human melanoma while suggesting actionable targets in both species.

## Author summary

Melanoma, a cancer arising from transformed melanocytes, commonly occurs in pet dogs. Unlike human melanoma, most often occurring in sun-exposed cutaneous skin, canine melanoma typically arises in sun-shielded oral mucosa. Canine melanoma clinically resembles human melanoma, particularly sun-shielded subtypes. However, canine melanoma genomics remain poorly defined. Similarly, although new treatments for human melanoma based on genomic understanding have shown improvements in outcomes for these patients, treatments for canine melanoma are limited and outcomes remain poor. Detailing the genomic basis of canine melanoma thus provides untapped potential for improving lives of pet dogs and helping to define canine melanoma’s role in comparative studies that also inform human melanoma understanding. To better define the genomic landscape of canine melanoma, we performed multi-platform characterization of 37 tumors. Our integrated analysis confirms that these tumors commonly contain mutations in canine orthologs of human cancer genes such as *RAS*, *MDM2*, and *TP53* alongside mutational patterns sharing important similarities with human melanoma subtypes. We have also found a new putative cancer gene, *PTPRJ*, frequently mutated in canine melanoma. These data will guide biologic and therapeutic studies in canine melanoma while broadly framing the utility of comparative studies of canine and human cancers.

## Introduction

Human melanoma is of increasing clinical concern. It is one of a few cancers with rising incidence, while five-year survival for patients with metastatic disease has until recently remained low (15–20%) due to a dearth of curative systemic therapies [1]. Discovery of frequent activating BRAF mutations in melanoma and treatment with selective inhibitors of this mutant kinase has led to dramatic responses in the setting of metastatic disease [2–4]. However, not all *BRAF*-mutant melanomas respond to targeted therapy and responses that do occur are often brief and followed by the emergence of drug-resistant disease [5]. Moreover, targeted treatment options in melanoma subtypes without activating *BRAF* mutations are limited. New treatment paradigms such as immunotherapy, drug combinations, and alternative dosing strategies may circumvent resistance and broaden the scope of precision medicine in melanoma [6–9], but rapid preclinical study of such regimens requires access to robust models that recapitulate complex tumor features such as intratumoral genomic heterogeneity and tumor-host interactions. Meanwhile, few animal models exist for uncommon molecular or histological melanoma subtypes such as *BRAF* wild-type (*BRAF*wt) or mucosal melanoma.

Naturally-occurring canine cancers are increasingly recognized as meeting a need for complex cancer models that develop gradually amidst interactions with the host stroma and immune system [10–16]. Spontaneous canine malignant melanomas, which are almost universally *BRAF*wt and for which the mucosal subtype is the most prevalent clinically significant form, may fill a specific gap in models of *BRAF*wt and rare histological melanoma subtypes [11]. Human mucosal melanoma is an aggressive histological subtype that is predominantly *BRAF*, *RAS*, and *NF1* wild type (Triple Wild Type or TWT) with occasional mutations in *KIT* or *NRAS*. It carries a five-year survival rate between 12.3% and 35.3% [17–26]. Study of this subtype is limited by its low prevalence, accounting for only 1–2% of human melanomas in the United States with as few as 1,500 cases per year [27]. On the other hand, canine malignant melanoma accounts for up to 100,000 yearly cancer diagnoses in the United States, occurring most commonly in the oral mucosa, but also arising in cutaneous and acral epithelium [28–31].

Canine malignant melanoma is highly prevalent, closely mirrors human melanoma clinically and pathologically, and is extremely aggressive, with median survival for oral cases being a mere 200 days[32–36]. However, little is known about its genetic etiology. It is predominantly *BRAF*wt with frequent copy number alterations of regions of canine chromosomes (CFA) 13, 17, 22, and 30, alongside frequent *MYC* amplifications and deletions of *CDKN2A*. Targeted sequencing studies, though limited, have shown that it infrequently bears alterations in other known drivers of human melanoma [32, 36–42]. It has been shown that CFA30 aberrations are characteristic of canine oral melanoma and complex copy number profiles on this chromosome homologous to the same profiles on human chromosome (HSA) 15 in human mucosal melanoma are suggestive of rearrangements that may drive this melanoma subtype [41]. Despite the very low prevalence of *BRAF* mutations, immunohistochemistry (IHC) has shown that the mitogen-activated protein kinase (MAPK) and/or phosphoinositide 3-kinase (PI3K) pathways are activated in 52–77% of cases [32, 36–40]. These data hint at underlying mutations driving these pathways that could guide future biological exploration and therapeutic development in the canine and human diseases.

We therefore set out to map the genomic landscape of canine melanoma using a combination of massively parallel whole genome sequencing (WGS), array-based platforms and targeted sequencing to identify somatic changes driving these cancers. Here we report the identification of recurrent inactivating mutations in the candidate tumor suppressor gene *PTPRJ* in addition to frequent *RAS* mutations, and mutually-exclusive *MDM2* and *TP53* alterations. We thereby define the genomic landscape of these cancers and identify similarities between melanoma subtypes across species while highlighting subtype-specific aberrations that may be used to guide future research.

## Results

### Patterns of mutation identified by whole genome analysis of canine melanoma

We undertook comprehensive analysis of acquired genetic alterations in a discovery cohort of seven melanomas and matched germlines from six dogs (two tumors were derived from one dog) using WGS for detection of subtle sequence alterations alongside long-insert WGS (LI-WGS, see Materials and Methods) [43] for sensitive detection of structural variants. We then performed copy number and targeted gene analyses in an additional 27 canine malignant melanoma tumors and three canine malignant melanoma cell lines (Table 1). Snap-frozen tumors (all primary tumors except one acral metastasis) and matching whole blood were collected through an IACUC-approved protocol at the Van Andel Research Institute (VARI) from dogs undergoing surgery at 21 specialty veterinary clinics located in 10 states (see Materials and Methods). Diagnosis of melanoma was confirmed by two independent board-certified veterinary pathologists (an on-site pathologist and BD) in addition to staining for three melanocytic differentiation markers where tissue was available (in 26 samples as indicated in S1 Table) [36, 44]. Diverse breeds are represented in this cohort with enrichment for Cocker Spaniels and Golden Retrievers (five dogs of each breed), an equal ratio of male and female dogs and a median age at resection of 11 years. Clinicopathologic characteristics for this cohort are described in S1 Table and S1 Fig.

**Table 1:** Summary of genomic analyses performed in canine melanoma.

Analysis platform	Type of alteration detected	Samples analyzed
**Discovery cohort**		
WGS	Point mutations, copy number, structural alterations	7 tumor and 6 matching normal samples
LI-WGS	Copy number and structural alterations	
mRNASeq	Expressed point mutations and transcript abundance	
aCGH	Copy number alterations	
SNP-A	Copy number alterations	
**Prevalence cohort**		
Targeted Sequencing	Point mutations	27 tumor and 11 matching normal samples, 3 cell lines
SNP-A	Copy number alterations	
Total distinct samples		34 tumor samples, 18 matching normals, 3 cell lines

WGS = whole genome sequencing; LI = long insert mRNASeq = messenger RNA sequencing

aCGH = array comparative genomic hybridization

SNP-A = single nucleotide polymorphism array.

For WGS and LI-WGS respectively a median of 38/11-fold sequence coverage and 209/155-fold physical coverage was achieved (S2 Table). Read alignment was performed using the canine reference genome CanFam 3.1 and stringent criteria were used to call somatic sequence variants intersecting Seurat v2.6, Strelka v1.0.13, and Mutect v1.1.4 (Materials and Methods). A total of 31,053 somatic single nucleotide variants (SNVs) and small insertions and deletions (indels) were found with a median of 4,223 genome-wide SNVs (range 1,880–6,342) and 316 indels (range 88–655) and a median mutation rate of 2.03 mutations per callable haploid megabase (range 0.97–3.14, Table 2). The genome-wide SNV spectrum showed C:G>T:A transitions to be most prevalent, at a median of 27.09% of total SNVs followed by T:A>C:G transitions (median of 21.19%) and C:G>A:T transversions (median 15.74%, S2 Fig). Despite the prevalence of C:G>T:A transitions, most occurred in CpG dinucleotides and were not enriched at dipyrimidines (median 22.5%). Therefore, a canonical UV signature was not present in any of these cases (S2 Fig) [45, 46]. We additionally looked for *TERT* promoter mutations, which have been reported in 71% of human cutaneous melanomas and are associated with UV damage [47], but no somatic mutations were found within one kilobase of the *TERT* transcription start site. The most common mutation overall was C:G>T:A in GCG trinucleotides (median 3.29%) followed by C>T in ACG (median 2.6%) and C>A in TCT (median 2.5%) (S2 Fig). No evidence of localized hypermutation (kataegis) was identified in these tumors [48].

**Table 2:** Summary of whole-genome analysis in canine melanoma discovery cohort.

Sample Information					SNVs			CNVs				SVs				
Sample	Tumor Type	Breed	Gender	Age at Diagnosis	SNV (n)	Indel (n)	Mut Rate	CNV (n)	CNV%	Amp (n)	Del (n)	SV (n)	CTX(n)	Inv (n)	Del (n)	Dup (n)
ND09-345	Mucosal	ECS	F	11	4223	264	2.03	41	0.4%	33	8	56	15	17	17	7
ND10-370	Mucosal	LR	M	10	6342	655	3.14	64	2.1%	23	41	65	9	22	21	13
ND10-376	Mucosal	CS	F	16	5085	344	2.48	4	0.3%	0	4	25	2	10	5	8
ND10-166	Mucosal	CS	M	14	3395	316	1.23	68	0.7%	61	7	34	2	11	12	9
ND10-361	Mucosal	CS	M	15	3029	88	1.42	5	0.0%	2	3	24	6	10	3	5
ND10-363	Acral	CS	M	15	4906	323	2.45	11	0.2%	2	9	9	0	2	5	2
ND10-441	Cutaneous	CS	F	11	1880	203	0.97	27	9.9%	0	27	39	8	12	12	7

SNV = somatic single nucleotide variant; Indel = insertion and deletion; Mut Rate = Mutation Rate (SNVs + Indels / Callable Mb); CNV = somatic copy number variant; CNV% = percentage of genome involved in CNVs; Amp = amplification-log ratio > = 2; Del = deletion-log ratio < = -0.6; SV = somatic structural variant from LI; CTX = inter-chromosomal translocation; Inv = inversion; Del = Deletion; Dup = duplication; ECS = English cocker spaniel; LR = Laborador retriever; CS = Cocker spaniel; F = female; M = male.

### Somatic coding mutations identified in canine melanoma

**Fig 1:**
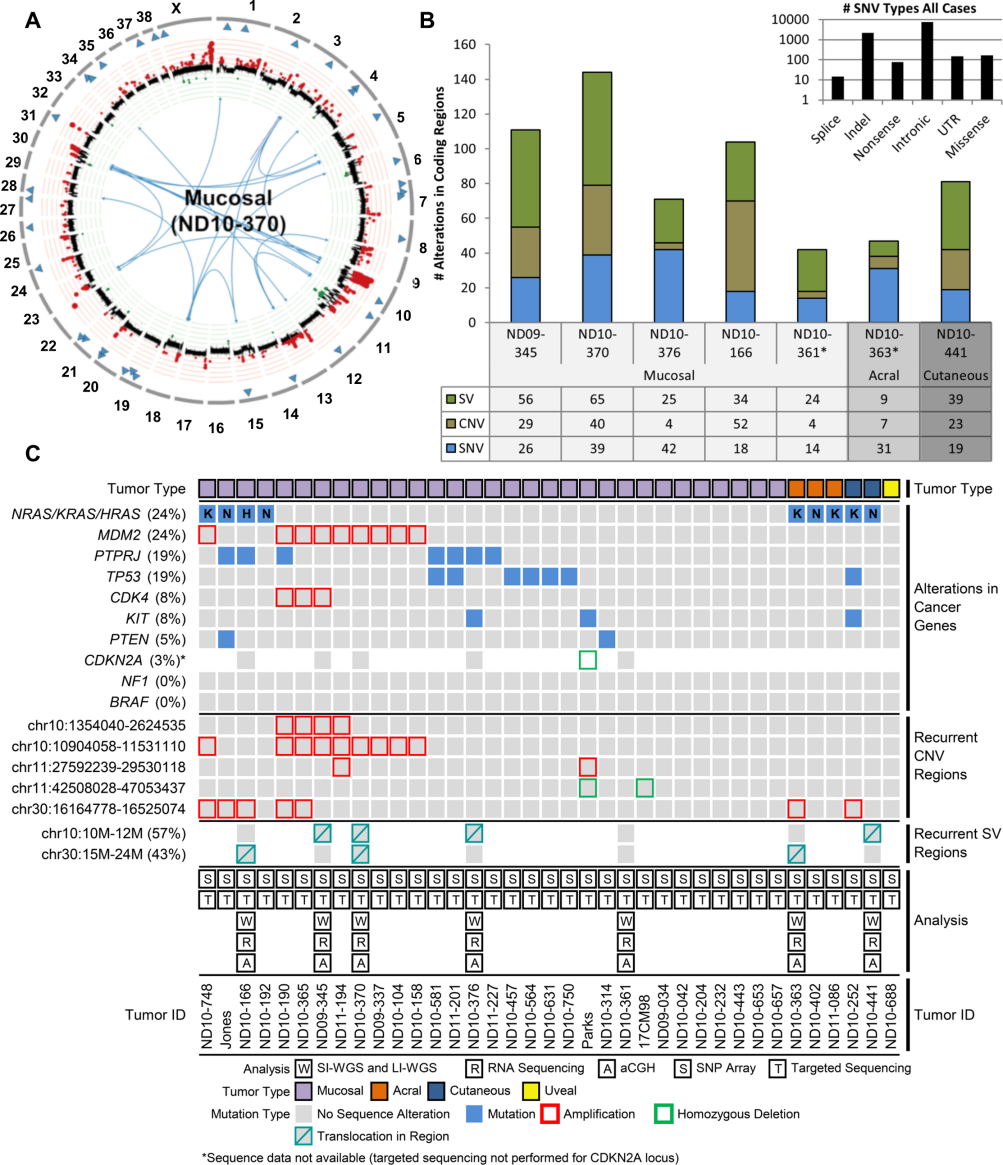
The mutational landscape of canine melanoma. (A) A representative Circos plot depicting coding SNVs, CNVs, and SVs in a single mucosal melanoma. Outer circle depicts canine chromosome number. Blue triangles are SNVs located within coding regions. The middle circle denotes CNVs with gains (in red) and losses (in green) according to the aberration amplitude. Blue lines transecting the plot show translocations. (B) Numbers and types of coding mutations identified by SI-WGS and LI-WGS in the discovery cohort. *ND10-361 and ND10-363 are independent primary tumors from the same dog. (C) Integrated genomic data is presented for 34 canine melanomas and 3 canine melanoma cell lines. Each column represents data from a single tumor. Indication of tumor type (mucosal, uveal, acral, and cutaneous) is displayed above annotation of recurrently-mutated and hallmark genes. Mutations identified by WGS, aCGH, SNP array, and targeted sequencing are presented in order of frequency as are recurrent CNV regions identified by SNP array and GISTIC as well as recurrent regions involved in translocations identified by LI-WGS. Genomic analysis annotation, tumor ID, and figure legend are presented at the bottom of the figure.

Tumors assessed by whole-genome analysis displayed an abundance of somatic structural variants (SVs) and copy number variants (CNVs), with a modest burden of SNVs in coding regions (Fig 1A and 1B). The landscape of somatic mutations in the full cohort of 37 tumors based on multi-platform analysis is shown in Fig 1C. Circos plots depicting somatic alterations in each tumor in the discovery cohort are shown in S3 Fig. Of the genome-wide SNVs described above, a median of 26 nonsynonymous (ns) single-base substitutions and indels occurred within coding regions (nsSNVs, range 14–42) with a median nonsynonymous: synonymous mutation ratio of 2.3 (range 1.9–3.9) (Fig 1B). We additionally performed RNA sequencing in this cohort, aligning with STAR2.4 [49], calling SNVs with HaplotypeCaller (GATK 3.3.0), and utilizing IGV to manually validate expressed sequence variants (Materials and Methods). Ninety-seven percent of nsSNVs (all but five) identified by WGS and genotyped on more than one sequencing platform were confirmed in at least one additional platform (S3 Table).

A number of mutations in orthologs of human cancer genes were present in a single tumor each. These genes include: *ATF6*, *EPAS1*, *FAT2*, *FAT4*, *FOXA3*, *FOXO1*, *GAB2*, *HRAS*, *KIT*, *KRAS*, *MMP21*, *NRAS*, *PBX1*, and *XPO1*. Although no recurrent SNVs were seen in the discovery cohort, three genes were mutated in two cases: *FAT4*, *LRFN2*, and *PTPRJ*. Of these, only *PTRPJ* was validated in multiple platforms in both cases. Both cases containing somatic *PTPRJ* mutations were mucosal (ND10-166 and ND10-376) and both putatively contained two hits. To determine the prevalence of mutations in a panel of genes whose orthologs are known to play a role in human melanomagenesis, as well as the *PTPRJ* gene mutated in two cases, we performed targeted Sanger sequencing of all protein-coding regions of *BAP1*, *BRAF*, *CDK4*, *GNA11*, *GNAQ*, *KIT*, *KRAS*, *MDM2*, *MITF*, *NF1*, *NRAS*, *PTEN*, *PTPRJ*, and *TP53* in the expanded cohort. *BRAF*, *CDK4*, *GNAQ*, *MDM2*, *MITF*, and *NF1* were all found to be universally wild-type whereas putative pathogenic mutations were discovered in *BAP1*, *GNA11*, *KIT*, *KRAS*, *NRAS*, *PTEN*, *PTPRJ*, and *TP53* as described below and in S4 Table.

### Somatic copy number and structural variants identified in canine melanoma

Somatic CNVs in the discovery cohort were identified by analysis of short-insert whole genome sequencing (SI-WGS) using established methods (Materials and Methods). A median of 27 focal CNVs (range 4–68), two focal amplifications with a log_2_ ratio ≥ 2 (range 0–61), and eight focal deletions with a log_2_ ratio ≤ 0.2 (range 3–41) were identified in the discovery cohort (Table 2 and S5 Table) comprising 0%-10% of the genome (Table 2). CNVs were additionally identified in this cohort utilizing Illumina CanineHD BeadChip Single Nucleotide Polymorphism (SNP) arrays and Agilent SurePrint G3 Canine Genome CGH microarrays as previously described [41, 50] (Materials and Methods) with a high platform concordance (S4 Fig). CNV analysis was then expanded to a total of 37 melanomas through SNP arrays in an additional 30 cases in the prevalence cohort (Table 1 and S5 Table). Altered regions were assessed by GISTIC [51] for statistically significant frequency and amplitude (G-score >1.0 and Q<0.05). Ten significant regions were identified including losses within CFA 1, 11, 15, and X, as well as gains in CFA10, 11, 13, 30, and X (S6 Table). Nine of 10 GISTIC regions contained genes and included gains in orthologs of the human cancer genes *MDM2* and *CDK4*. Additional cancer driver alterations (homozygous deletions of tumor suppressor genes or focal amplifications of oncogenes) included *CDKN2A* homozygous deletion (3%) and *KIT* focal amplification (8%) (S7 Table).

Somatic SVs including translocations, inversions, and duplications, were identified in the discovery cohort, based on calls from Delly v0.7.6 [52] in LI-WGS (Materials and Methods). Between 9 and 65 predicted SVs were identified in each tumor (median 34) and were predominantly inversions (Table 2 and S8 Table). No recurrent rearrangements were present. Notable alterations in human cancer gene orthologs impacted by SVs in single cases include an *ARHGEF12* inversion, a *BIRC3* inversion, a *CLPTM1L-TERT* translocation, a *DDIT3* inversion, a *MYO5A* translocation, and a *TCF12* inversion. However, two regions of CFA10 and 30 were found to contain somatic SVs in two or more tumors. CFA10 rearrangements occurred in five of seven cases, four of which bore alterations in the region spanning 10–12 Mb (also a significant GISTIC region from CNV analysis). CFA30 SVs were also present in three tumors with alterations occurring within a region spanning 15–24 Mb (also encompassing a GISTIC region) in each case. Complex chromosomal rearrangements reminiscent of chromothripsis were observed in four tumors (ND09-345, ND10-370, ND10-361, and ND10-441), with chained or clustered breakpoints localized to a subset of chromosomes in regions that also contained copy-number oscillations [53] (S3 Fig).

### *BRAF*, *RAS*, *NF1*, and *KIT* mutations

**Fig 2:**
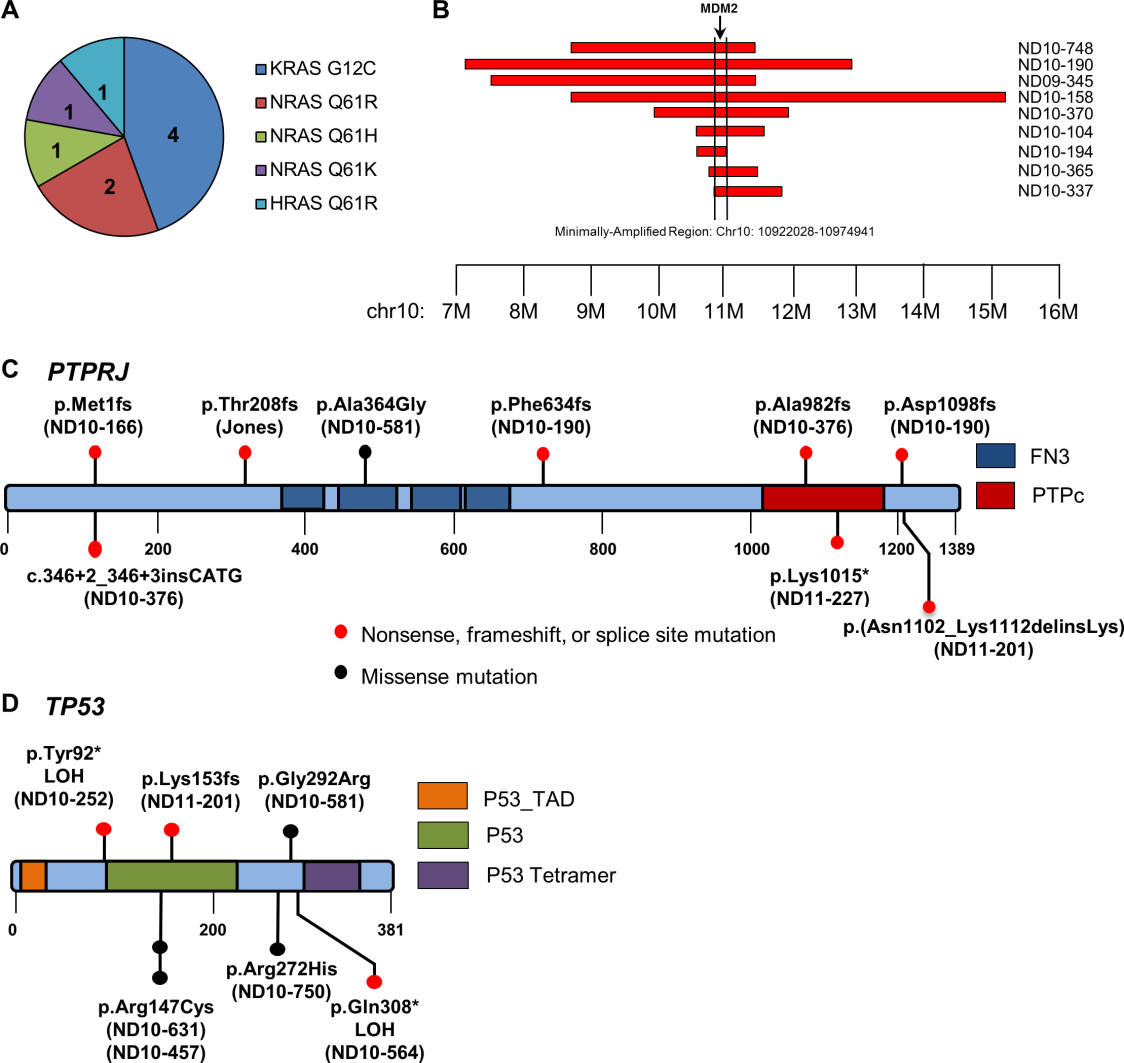
Recurrent somatic alterations in canine melanoma. (A) Distribution of RAS mutations within the cohort of 37 samples (n = 9). (B) Recurrently amplified region of CFA10 found in nine tumors, which is defined by the minimal region surrounding *MDM2*. (C) Location of potentially deleterious mutations present in the putative tumor suppressor *PTPRJ* found through Sanger sequencing of the coding sequence of each tumor. (D) Individual mutations and their locations within *TP53*.

Approximately 90% of human cutaneous melanomas are driven in part by *BRAF*, *RAS*, *NF1*, and *KIT* mutations that confer constitutive mitogenic signaling through the MAPK pathway [24, 45, 54]. However, these alterations are far less common in human mucosal and acral melanomas [20, 22, 23, 55–57]. No somatic alterations in *BRAF* were identified within any platform in our canine melanoma cohort. However, *RAS* family members, whose protein products are predicted to share 100% sequence identity with their human orthologs, were the most commonly mutated genes in aggregate, occurring in 24% of cases in human-conserved hotspots (Figs 1C and 2A). *NRAS* codon 61 (Q61R/H/K) and *KRAS* codon 12 (G12C) mutations occurred each in four cases while a single case bore an *HRAS* Q61R mutation (nine total RAS mutations). All three acral and two cutaneous cases bore *NRAS* or *KRAS* mutations, while only 4/31 (13%) of mucosal cases bore an *NRAS*, *KRAS*, or *HRAS* mutation. Although *NF1* copy number losses occurred in six cases, no homozygous deletions or truncating mutations were identified (S7 Table). *KIT* mutations were present in one cutaneous and two mucosal tumors (S3 and S4 Tables). In the cutaneous case, the mutation results in a glutamine (Q) to arginine (R) change in codon 396, notably a site of variation between canine and human orthologs, a change that is not predicted to be damaging by PROVEAN, and may constitute a germline SNP, but germline DNA was not available in this case [58]. *KIT* mutations in the mucosal cases included an in-frame deletion of amino acids 560–562, a likely damaging mutation in a commonly mutated region of the human ortholog, as well as an aspartic acid (D) to valine (V) change in codon 815 corresponding to the most common hotspot D816V mutations occurring in the kinase domain of *KIT* in human cancers (S5 Fig) [59]. Copy number gains encompassing *KIT* were also present in 10 samples (eight mucosal, one acral, and one cutaneous–Jones, 17CM98, ND10-104, ND10-158, ND10-365, ND10-370, ND10-376, ND10-361, ND10-363, and ND10-441), although no focal amplifications were identified (S7 Table).

### *PTPRJ* mutations

The most commonly mutated gene in this cohort was the putative tumor suppressor gene *PTPRJ*, not previously shown to have frequent inactivating point mutations in cancer (Figs 1C and 2C). PTPRJ (also known as density-enhanced phosphatase 1 (DEP-1) or CD148) is a protein tyrosine phosphatase receptor originally discovered by virtue of its overexpression in dense cultures of human lung fibroblasts [60]. It has since been shown to be frequently involved in allelic loss or loss of heterozygosity (LOH) in human cancers and mouse models [61, 62] and to potentially play a role in oncogenesis in diverse cancer types, but somatic homozygous deletions or truncating mutations have yet to be described in cancer from any species and its tumor suppressor status remains controversial [63–71]. Canine and human orthologs share 70% sequence identity with a highly conserved C terminus containing the protein tyrosine phosphatase catalytic domain that is nearly 100% identical between species (S6 Fig). Sequencing of *PTPRJ* across all 37 tumors revealed nine mutations in seven cases (all mucosal), comprising 19% of all tumors and 23% of mucosal cases. Six frameshifts or stop gains were discovered in addition to two splice site mutations, a C-terminal 10-amino acid deletion, and a single predicted damaging missense mutation. Two cases–ND10-190 and ND10-376 –contained two mutations each, consistent with putative bi-allelic inactivation of a tumor suppressor gene. Further, LOH was evident by analysis of adjacent SNPs in WGS data in case ND10-166 bearing the M110fs mutation (S9 Table). Consistent with this finding, the *PTPRJ* frameshift in the ND10-166 tumor occurred at an allele ratio of 61% in DNA and 100% in RNA. Finally, PTPRJ transcript was observed in RNAseq data from the two *PTPRJ*-mutant tumors profiled by WGS and RNAseq (270.21 Fragments Per Kilobase of transcript per Million mapped reads (FPKMs) in ND10-166 and 92.37 FPKMS in ND10-376) as shown in S7 Fig. ND10-376, containing two somatic *PTPRJ* mutations (a frameshift and a splice site mutation) and 92.37 FPKMs, bore the lowest transcript abundance among all seven profiled tumors. Median PTPRJ FPKMs for the five PTPRJ wild-type tumors was 171.76 (range 92.37–512.25).

### *MDM2* amplifications and *TP53* mutations

Inactivation of the p53 network is a critical step in tumorigenesis in nearly all cancers [72]. Both truncating *TP53* mutations and amplifications of *MDM2*, a negative regulator of p53, are key routes to p53 inactivation [73]. Although *TP53* mutations and *MDM2* amplifications in human melanoma are less common [23–25, 45, 54, 56], 16/37 (43%) of the cases in our cohort of canine melanoma bore focal amplifications of *MDM2* or truncating *TP53* mutations (Fig 1C). A recurrent focal amplification on CFA10 was identified by whole genome analysis in three of seven tumors in the discovery cohort with extended SNP array analysis in the prevalence cohort revealing an additional eight tumors bearing these amplifications (minimal region 10.9–11.8 Mb) (Figs 1C and 2C). In total, 11/38 cases (29%) bore this amplification involving seven genes, with *MDM2* being the likely amplification target (Fig 2B). All such amplifications occurred in mucosal melanomas (11/31, 35%). *CDK4*, a cancer gene 10 Mb proximal to *MDM2* in both human and canine genomes and often the target of bipartite amplification alongside *MDM2* [74, 75], was co-amplified in three of these cases. Identification of focal MDM2 or CDK4 amplification in the WGS- and RNA-sequenced cohort coincided with high transcript abundance for these genes relative to their wild-type counterparts (S7 Fig). MDM2 FPKMs were 357.48 and 331.21 for the amplified cases (ND09-345 and ND10-370) relative to a median of 67.02 (range 37.57–82.26) for wild-type cases. CDK4 FPKMs were 2,730.13 for the amplified case (ND09-345) versus a median of 201.24 (range 69.87–471.2 for the wild-type cases). Additionally, twenty tumors were additionally assessed for MDM2 expression by IHC (S10 Table and S8 Fig). Three of five cases with *MDM2* focal amplifications also showed prominent MDM2 staining while no cases lacking *MDM2* amplifications were positive by IHC.

We additionally discovered seven tumors with mutations in *TP53* whose protein product shares 80% identity with its human ortholog (S9 Fig). Three of these mutations were truncating–a homozygous T90X in ND10-252, heterozygous K151fs in ND11-201, and a heterozygous Q306X in ND10-564 (Fig 2D and S4 Table). Of the three missense mutations, R145C and R270H were predicted to be damaging. R145C occurred in two tumors and R270H in a single tumor, with both mutations confirmed somatic through analysis of matched germline DNA. Codon 270 in canine *TP53* is homologous to codon 282 in human *TP53*, the fifth most common hotspot for mutations in human cancer[59]. The missense G290R variant is a likely SNP. It occurs in a tumor for which matched germline DNA is unavailable and it is predicted to be neutral, although it has not been previously described [76–78]. In keeping with findings in other cancers, no sequence mutations were present in *MDM2* and *MDM2* amplifications were mutually exclusive with *TP53* mutations. Further, *TP53* and *MDM2* alterations were mutually exclusive with *RAS* mutations in all but one case (ND10-748, Fig 1).

### Pathway dysregulation in canine melanoma

Common genomic subtypes of human cutaneous melanoma (*BRAF*, *RAS* (N/H/K), and *NF1* in 90% of cases) that engage oncogenic signaling through the MAPK pathway are less common in human non-cutaneous melanoma and in canine malignant melanoma (24% of cases here, Fig 1C). Therefore, to undertake unbiased identification of pathways contributing to canine melanomagenesis, we performed pathway analysis using WGS data from the discovery cohort. We generated a list of all genes bearing nonsynonymous mutations, lying within chromosomal breakpoints or significant CNV regions from GISTIC (n = 1047) in order to determine enrichment of these mutated genes within specific KEGG and Reactome pathways (Materials and Methods) [79–81]. Network analysis of the affected genes identified 97 pathways with significant Benjamini-Hochberg corrected *P*-values (S11 Table). The most significantly enriched pathways were Insulin Receptor Substrate (IRS)-mediated signaling, and IRS-related events, for which 23% (19 genes) of the pathway members are mutated in this cohort. Notably, these pathways converge on MAPK and PI3K mitogenic signaling and contain core pathway members such as *FGF*s, *EIF4G1*, *HRAS*, *KRAS*, *NRAS*, and *RPTOR*. Indeed the majority of the enriched pathways contain members of MAPK, PI3K, or growth factor receptor signaling (e.g. PI3K cascade P = 0.002, mTOR signaling P = 0.008, signaling by Rho GTPases P = 0.012, VEGF signaling P = 0.017, RAF activation P = 0.017, melanoma signaling P = 0.021, RAS signaling P = 0.031, and MEK activation P = 0.036) and, in many cases, intersections with MDM2 signaling.

## Discussion

Melanoma is a clinically significant disease in dogs, the study of which holds untapped potential for developing clinical approaches to improve the lives of pet dogs while also informing human melanoma biology and treatment. Few treatment options are available for locally advanced or metastatic canine melanoma in part because the molecular etiology is still largely unknown. Similarly, limited molecular understanding of rare sun-shielded and *BRAF*wt subtypes of human melanoma has constrained clinical innovation. In order to identify the molecular alterations underlying canine melanoma, we undertook a comprehensive multi-platform genomic investigation. Our integrated analysis confirms that although these tumors are driven by mutational landscapes distinct from those in human cutaneous melanoma, they share important similarities with *BRAF*wt and rare histological subtypes of human melanoma. These data not only guide biological and therapeutic studies in canine melanoma, but they also lend further support for the use of the naturally occurring canine model in comparative studies of human cancers.

This study builds on knowledge of the cytogenetic landscape of canine melanoma [41] to provide a comprehensive view of numbers and types of somatic coding mutations in this cancer. Given the dearth of genomic data for canine melanoma, we focused overall on collecting primary tumors from diverse breeds. While this study was not sufficiently powered to draw conclusions regarding breed associations with somatic mutations, it is nonetheless important to consider potential associations between breed and somatic mutational landscapes, particularly because such associations have been shown to occur in other canine cancers such as lymphoma [82]. Several breeds have been suggested to be at increased risk for malignant melanoma, particularly breeds with heavily pigmented skin or oral mucosa such as Cocker Spaniels, Schnauzers, Scottish Terriers, Poodles, Chow Chows, and Golden Retrievers [83]. Our WGS discovery cohort primarily consisted of Cocker Spaniels (four Cocker Spaniels, one English Cocker Spaniel, and one Labrador), a breed reported to be at higher risk of oral melanoma, but our extended cohort then included targeted sequencing of 13 melanoma hallmark genes (as well as *PTPRJ*, which was the only additional recurrently mutated gene in the WGS cohort) and copy number assessment from SNP arrays across 20 total breeds. Given that our WGS cohort was predominantly Cocker Spaniel, it is possible that other recurrent, breed-specific somatic SNVs in non-melanoma-hallmark genes could exist that were not captured here. Thus, future expanded study of breed-specific cohorts will be critical for further understanding the role of germline variation in shaping somatic cancer landscapes across species. It will also be important to further define subtype differences in expanded cohorts of canine acral and cutaneous tumors as well as benign and precursor lesions.

**Fig 3:**
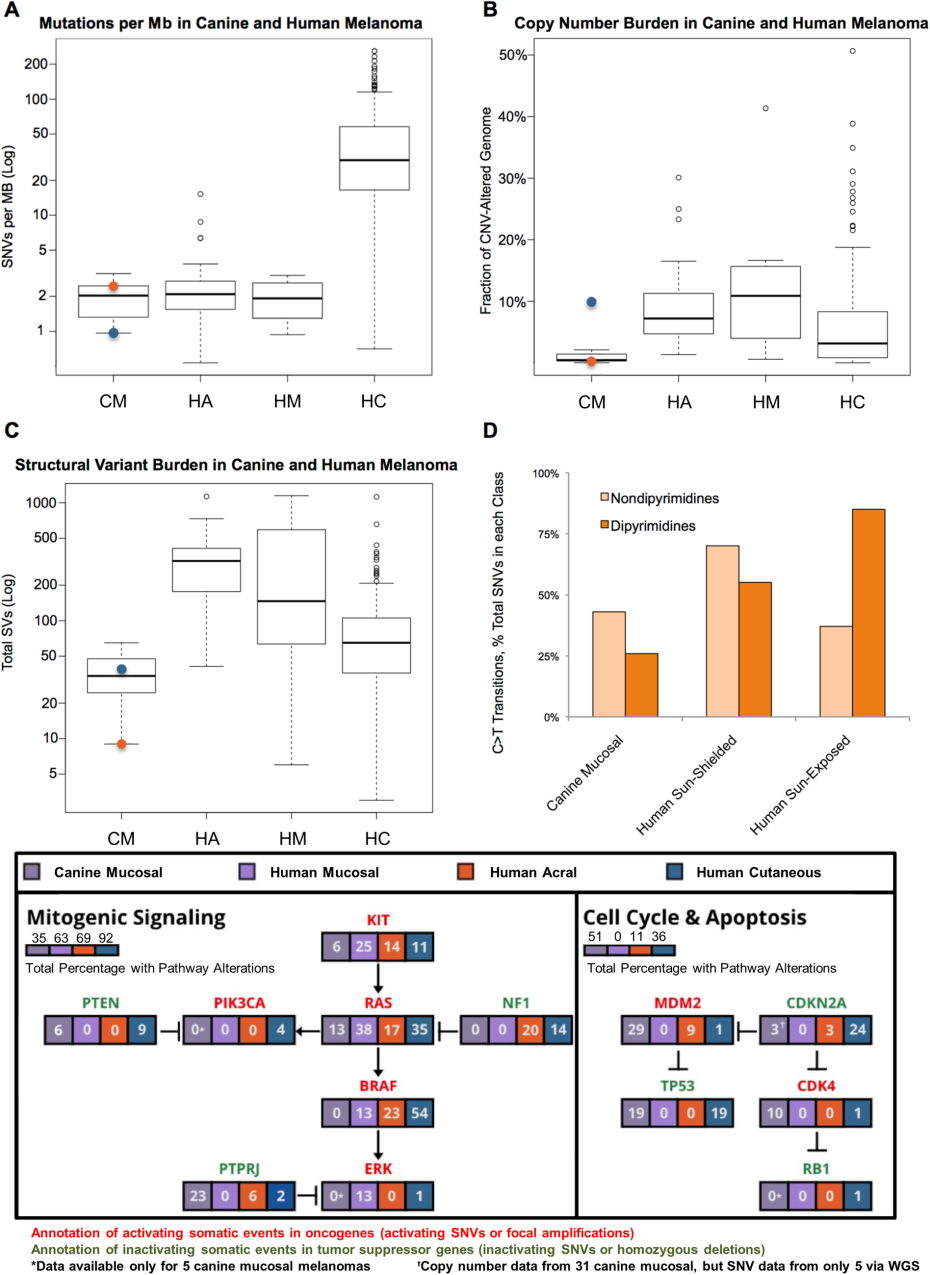
Key deregulated pathways in canine and human melanoma. (A) Mutation rate in canine and human melanoma subtypes is shown as somatic SNVs per DNA Mb based on WGS in our discovery cohort compared to WGS data from 140 human cutaneous, 35 acral, and 8 mucosal melanomas (Hayward *et al*., 2017). CM = Canine mucosal, HA = Human acral, HM = Human mucosal, and HC = Human cutaneous melanoma. Orange and blue dots in the CM plot represent the individual acral and cutaneous subtypes, respectively, in our discovery cohort. (B) Fraction of copy-number-altered genome in canine melanoma and human melanoma sequencing cohorts. (C) Total number of structural variants identified in canine and human melanoma sequencing cohorts. (D) Comparison of C>T transitions in the major melanoma types in dipyrimidine versus non-dipyrimidines. (E) Overall frequency of mutations in key melanoma pathways in our full cohort of 31 mucosal tumors compared to WGS in other subtypes from Hayward *et al*., 2017.

Overall, the genomic landscapes of human melanoma vary by anatomic site and degree of sun exposure [22, 26, 57]. Cutaneous sun-exposed melanoma is characterized both by high point mutation frequencies linked to UV damage [45] and also only modest burdens of structural variation. In contrast, sun-shielded and non-cutaneous melanomas harbor a low point mutation, but high structural mutation burden. Here, we establish that the canine malignant melanoma genome landscape resembles that reported in human sun-shielded melanoma. Canine melanoma of all subtypes in our discovery cohort is likely sun-shielded, including cutaneous tumors which occur in densely hair-bearing skin, although cropping or shaving during summer months may in some cases increase UV exposure. In keeping with this status, WGS in these two canine cutaneous malignant melanoma cases provides a deep view of their genome-wide mutation burden revealing low point mutation frequencies (median 2.03 somatic mutations per Mb) similar to that seen in human acral and mucosal melanoma WGS data from Hayward *et al*. 2017 (Fig 3A) [26]. Although we only profiled two such cases and larger cohorts are needed, a low point mutation burden relative to human sun-exposed melanoma has potential bearing on expected responses to immunotherapy such as anti-CTLA4 and anti-PD1 checkpoint blockade. Numerous studies have shown a clear positive correlation between mutation burden, abundance of neoantigens, and clinical benefit in human melanoma and other cancers [84, 85]. Nonetheless, other molecular determinants of response to immunotherapy exist beyond simply mutation burden and the activity of such agents in canine malignant melanoma remains to be determined. Notably, CNV and SV burden from our WGS in canine malignant melanoma was markedly lower than all subtypes as described in Hayward *et al*. (Fig 3B and 3C) [26].

WGS additionally provides a deep view of genome-wide mutation signatures. High point mutation burden in sun-exposed cutaneous melanoma is understood to result from UV-induced over-representation of C>T transitions occurring in dipyrimidines versus non-dipyrimidines. UV-induced C>T mutations occurring in dipyrimidines comprise a low proportion of total SNVs in our cohort (25%), reflective of human sun-shielded cutaneous, mucosal and acral melanoma, in contrast to 85–90% of C>Ts occurring in dipyrimidines in human sun-exposed melanoma (Fig 3C) [24, 26, 45, 55, 56, 86]. This lends support for a non-UV etiology of canine melanoma.

The genome-wide SNV spectrum further revealed that C>T transitions in CpGs were the most common sequence alterations (S2 Fig). These mutations correlate with age in human cancers and are due to spontaneous deamination of 5-methylcytosine [46]. Enrichment for these mutations in canine melanoma is not surprising given that the largest risk factor for cancer in humans and dogs is biological (not chronological) age [87–92] and that the mean age of these dogs at the time of surgical resection was 13 years (range: 10–16). Relative to the average number of human somatic mutations, these data provide further evidence that not only cancer incidence, but also mutational burden increases with biological, rather than chronological, age [93]. Commonly observed mutational patterns in human melanoma such as kataegis were not observed, although four tumors exhibited clustered or chained translocations suggestive of breakage-fusion-bridge events due to telomere crisis or of chromothripsis, in which one or a few chromosomes undergo punctuated shattering and reassembly events [53]. Such events have been linked to poor outcome in human melanoma [94] and may be enriched in tumors with p53 dysfunction or those that lack means to extend telomeres [95, 96]. Notably, we show here that *MDM2* and mutually exclusive *TP53* alterations are common in canine melanoma. Similarly, inactivating p53 mutations have been found in human mucosal and acral melanoma, suggesting p53 pathway dysregulation may be crucial in non-UV induced melanoma development. Further, UV-induced *TERT* promoter mutations are common in human cutaneous melanoma, and, although they are rare in sun-shielded subtypes, these subtypes have been shown to bear enrichment for other types of mutation that drive TERT overexpression such as SVs and CNVs [57]. The cutaneous tumors in this cohort do not bear somatic *TERT* promoter mutations or other known genetic lesions that would enable telomere extension. Thus, telomere crisis and the survival of structurally aberrant genomes may play a significant role in the molecular etiology of canine melanoma.

Our comprehensive analysis of canine melanoma reveals that most canine melanomas bear a low coding mutation burden and are also less structurally complex than human melanoma. Two WGS approaches coupled with array-based platforms have enabled deep interrogation of these changes, complementing recent cytogenetic analyses of this tumor type [41]. Significant copy number gains on CFA10 and 30 that have been reported as a defining signature of these lesions are recapitulated in this dataset (S6 Table). Our multi-platform approach was also able to further elucidate complex chromosomal rearrangements present in these regions. Both regions are involved in multiple intra- and inter-chromosomal structural events across this cohort (S8 Table). Additionally, focal amplification of the CFA10 10-12MB region encompasses *MDM2*, a gene which is known to drive human cancers and is currently being explored as a drug target in TP53 wild type tumors [97]. CNVs associated with canine melanoma also include gain of CFA13 and loss of CFA22. While not statistically significant via GISTIC in this cohort, both events are present in individual samples. Overall, extensive copy number and structural variation suggest high levels of large-scale chromosome instability, i.e. gain and loss of whole chromosomes or chromosome arms. Intriguingly, mutually exclusive focal amplification of *MDM2* or inactivating mutation in *TP53* have been shown to be enriched in *BRAF*-, *NRAS*-, and *NF1*-wild-type human melanoma, although human *TP53*-mutant melanomas tend to also display higher mutation burden and presence of C>T transitions [98]. Taken together the high degree of structural complexity, the lack of *TERT* mutations (barring one putative translocation) or telomere-lengthening mechanisms, and the frequency of *MDM2/TP53* mutations all suggest that chromosome instability plays a key role in canine melanomagenesis.

In the discovery cohort, putatively pathogenic somatic mutations in orthologs of human cancer genes were present in a single tumor each including *ATF6*, *EPAS1*, *FAT2*, *FAT4*, *FOXA3*, *FOXO1*, *GAB2*, *HRAS*, *KIT*, *KRAS*, *MMP21*, *NRAS*, *PBX1*, and *XPO1* (S3 Table). Of the 14 melanoma hallmark genes evaluated in the prevalence cohort (including *PTPRJ*), an additional 24 putatively pathogenic somatic mutations were identified in seven genes–*NRAS*, *TP53*, *PTPRJ*, *KIT*, *KRAS*, *GNA11*, and *BAP1* (S4 Table). Overall, across discovery and prevalence analyses, RAS gene family members were the genes most commonly bearing somatic SNVs, occurring in 24% of cases (Figs 1C and 2A), followed by *TP53* and *PTPRJ* mutations each in 19% of cases, *KIT* in 8% and *PTEN* in 5%. Combined, these mutations most commonly impact proliferative and cell cycle/apoptosis pathways in patterns that display both key similarities and differences with human melanoma subtypes [26] (Fig 3D). For example, despite an absence of *BRAF* and lower abundance of *RAS* and *KIT* mutations in canine versus human mucosal melanoma, these tumors display likely MAPK activating events in 35% of cases. Further, canine mucosal melanoma displays a higher burden of cell cycle and apoptotic events (51%) than all subtypes from the Hayward comparator human melanoma cohort assessed here due largely to enrichment for mutually exclusive *MDM2* and *TP53* mutations in canine mucosal melanoma. However, these mutations are common in human cutaneous melanoma (ranging from 36% of cases in the Hayward comparator cohort to 62% in the TCGA cutaneous melanoma study [98]). Some of these differences are likely due to the still small sizes of non-cutaneous human and canine melanoma cohorts and the need for greater resolution, particularly across different anatomic sites. For example, all but two of the canine mucosal cases described in this study originate from the oral cavity, whereas the eight human mucosal comparators are largely vulvar (3) or from nasal cavity (2). Thus, an ongoing need exists for broader profiling of these melanoma subtypes by anatomic site. Overall, however, these findings nonetheless suggest that both MAPK pathway inhibition (e.g. via MEK inhibitors) and p53 pathway inhibition (e.g. via MDM2 inhibitors) are important therapeutic axes for development in canine melanoma just as they are in human [38].

The oncogenic MAPK pathway is critically important in many cancers given its central role in conveying extracellular signals to the nucleus in order to regulate cancer hallmarks including proliferation, invasion, metastasis, and angiogenesis. The majority of human cutaneous melanomas are driven in part by constitutive activation of the MAPK pathway through mutation of genes such as *BRAF*, *NRAS*, *NF1*, *KIT*, *GNAQ*, and *GNA11*, often in a mutually exclusive pattern [99]. The high frequency of these mutations has motivated the TCGA classification of these tumors according to MAPK mutation status: *BRAF* (~50% of cases), *RAS* (~30%), *NF1* (~15%), and TWT (~10%) [98]. These genomic categories are correlated with clinical, pathological, molecular, and biological features of melanoma and thus may comprise distinct subtypes. However, less common histological subtypes of melanoma such as mucosal, acral, and uveal melanoma bear unique mutation spectra that are not uniformly centered on canonical activating mutations in the MAPK pathway. Correspondingly, it has been shown that *BRAF* mutations are exceedingly rare in predominantly oral canine malignant melanoma and, to date, few alterations in other MAPK members have been discovered. These findings were recapitulated in our cohort, which showed no canonical *BRAF* or *NF1* mutations. Nonetheless, MAPK and/or PI3K signaling have been shown to be activated in nearly all cases [100]. Additional mutations impacting the MAPK and PI3K pathways include three *KIT* mutations, two *PTEN* mutations, and one *GNA11* mutation. In total, 35% of mucosal and 43% of all canine melanomas bear an alteration impacting the MAPK pathway (Figs 1C and 3D). Prior to our studies described here, the mutations underlying such activation have remained largely unknown.

Here we show a complete absence of somatic *BRAF* mutations (SNVs, CNVs, or translocations encompassing the *BRAF* locus) in canine malignant melanoma in keeping with prior studies [32, 37, 41, 101]. We also did not uncover truncating SNVs in or homozygous deletions of *NF1*. A higher proportion of our cohort bear *RAS* mutations than the 6–13% previously described [32, 100], although prior studies have focused almost exclusively on *NRAS* exons one and two. All three major RAS family members are highly conserved (100% protein identity) between canine and human. In humans, of these family members, malignant melanomas predominantly bear *NRAS* mutations with only very rare *KRAS* and *HRAS* mutations. In our cohort, we found four *NRAS* codon 61 alterations (11%), four *KRAS* G12C mutations and one *HRAS* Q61R mutation. Further, four of these RAS alterations (two *NRAS*, one *KRAS*, and one *HRAS* mutation) occur in mucosal tumors, a frequency of 13% in this subtype. However, in our cohort all three acral tumors and both cutaneous tumors had detectable *RAS* alterations (three *KRAS* and two *NRAS* mutations). This unusual pattern of *RAS* mutation in canine melanoma may reflect important differences in biological, tissue, and species specificities of RAS family members.

These data point to the genomic lesions underlying MAPK and PI3K activation in a substantial proportion of canine melanomas, and to subtle genetic differences in disease subtypes within and across species. Most striking is the discovery of a putative novel tumor suppressor gene, *PTPRJ*, a receptor-type protein tyrosine phosphatase, which has been genetically and functionally implicated in cancer [61, 62], but for which clear genetic mechanisms of inactivation have yet to establish its definitive role as a canonical tumor suppressor gene. *PTPRJ* consists of an extracellular domain with eight fibronectin III motifs, a transmembrane domain, and an intracellular catalytic domain. It was originally cloned from HeLa cells and characterized by overexpression and hyper-activation in dense cultures of fibroblasts, by regulation of contact inhibition, and by its role in regulation of cancer cell proliferation and invasion [60, 102–107]. Early genetic studies of quantitative trait loci for mouse cancer susceptibility with homologous regions in human cancers pointed to recurrent *PTPRJ* deletions, LOH, and missense mutations in small cohorts of colorectal (49%), lung (50%), and breast (78%) carcinomas in addition to a correlation between *PTPRJ* LOH and colorectal cancer progression [61, 62]. Additional sequencing studies in larger cohorts have identified nonsynonymous SNPs in the extracellular fibronectin repeats associated with risk of developing thyroid, colorectal, head and neck squamous cell, and esophageal cancers [67, 70, 108–110]. More recently, a subclonal K1017N missense mutation in the non-catalytic cytoplasmic domain of PTPRJ was identified in a primary breast tumor with significant enrichment in a brain metastases and patient-derived xenograft [111]. PTPRJ substrates that may mediate its tumor suppressive potential include ERK1/2, Akt, various receptor tyrosine kinases, and Src kinases [42, 112–116]. However, *Ptprj* knockout mice have normal development with no cancer predisposition and thus inactivation of this gene does not appear to be sufficient to induce tumorigenesis[65]. Across all TCGA studies published to date (10,951 cases from 33 tumor types in the TCGA PanCancer Atlas accessed via cBioPortal), the frequency of somatic *PTPRJ* point mutations and/or deep deletions is low– 211/10,951 (1.9%, S12 Table) [117, 118]. Only 21 somatic *PTRPJ* mutations are present in the TCGA human cutaneous melanoma data set consisting of 363 cases (a single homozygous deletion, five truncating mutations, and fifteen missense mutations). However, a related receptor-type protein tyrosine phosphatase, *PTPRD*, is thought play a role in regulation of STAT3 signaling and has been frequently implicated as a tumor suppressor in human cancers through inactivating somatic mutation, focal deletion or methylation in glioma, melanoma, neuroblastoma, colorectal, liver, head and neck, and lung cancers [119–122]. In human cutaneous melanoma, *PTPRD* is deleted or truncated in 9–12% of cutaneous cases, but has not been determined to occur at high frequency in rare histological subtypes [50, 55, 56, 120, 123].

Here, we present the first report of recurrent somatic truncating mutations in *PTPRJ* in a naturally occurring cancer. We have discovered seven cases (19%) of canine melanomas bearing somatic *PTPRJ* mutations. Canine and human PTPRJ orthologs share 70% sequence identity with a highly conserved C-terminus containing the protein tyrosine phosphatase catalytic domain (S6 Fig). Sequencing of *PTPRJ* across all 37 tumors revealed nine mutations in seven cases (seven mucosal and one uveal) comprising 19% of all tumors and 23% of mucosal cases. Six frameshifts or stop gains were discovered in addition to one splice site mutation, a C-terminal 10-amino acid deletion, and a single predicted damaging missense mutation. Two cases–ND10-190 and ND10-376 –contained two mutations each, consistent with bi-allelic inactivation of a tumor suppressor gene. Further, LOH was evident by analysis of adjacent SNPs in WGS data in case ND10-166 bearing the M110fs mutation (S9 Table). Although regional LOH on chromosome 18 was observed by SNP array in three of six cases bearing single mutations in *PTPRJ*, these regions were not observed to directly overlap the coding region of *PTPRJ*. Overall, the enrichment for PTPRJ truncating mutation in canine malignant melanoma bears intriguing implications both for a previously underappreciated role for this gene in human melanoma (e.g. through as-yet understudied roles for hemizygous deletion [124] and/or epigenetic modifications) and for the possibility of unique mechanisms of tumorigenesis across species.

Through deep integrated genomic analysis combining WGS, LI-WGS, RNA sequencing, aCGH, SNP arrays, and targeted Sanger sequencing we have determined that canine melanoma is driven by frequent dysregulation of MAPK and cell cycle/apoptosis pathways and, in some cases as is seen in our WGS cohort of predominantly Cocker Spaniels, extensive chromosomal instability. In keeping with prior comparative melanoma studies that have incorporated histology, targeted sequencing, and aCGH [32, 36, 38, 41], this work highlights the striking resemblance of canine malignant melanoma to *BRAF*wt subtypes of human melanoma. Finally, we have additionally discovered a putative novel tumor suppressor that may reflect unique species-specific biology and/or may highlight a tumor suppressive axis more subtly altered and as-yet underappreciated in human melanoma. This work bears immediate relevance for development of improved diagnostic and treatment approaches in canine malignant melanoma and provides further evidence to credential the naturally occurring canine melanoma model for study of relevant genomic subsets of human melanoma.

## Materials and methods

### Ethics statement

Samples were obtained under institutional review protocols at the Van Andel Research Institute in collaboration with local speciality veterinary clinics (protocol #08-06-14).

### Clinical samples, histopathology and sample assessment

Tumors and whole blood were obtained from 36 dogs recruited from 21 veterinary specialty centers in 10 states (AZ, CA, FL, IL, MA, MI, OH, TX, VA, WI) under VARI IACUC and ethical review (protocol #08-06-14). Material was collected at surgery and snap frozen in optimal cutting temperature (OCT) compound. Patient matched control DNA was obtained from peripheral blood mononuclear cells. Each resected tumor was evaluated by an on-site board-certified veterinary pathologist and then by BD to estimate tumor content and extent of tissue heterogeneity. Diagnosis of malignant melanoma was histologically confirmed according to criteria defined by the American College of Veterinary Pathologists in addition to criteria recently established by comparative analyses of canine and human melanoma focusing on architecture, pigmentation, and the presence of differentiation markers [32, 100, 125].

### Immunohistochemistry

Two tissue microarrays (TMAs), designated Dog MEL A TMA and Dog MEL B TMA, consisted of 96 individual dogs and 131 tissue samples placed in duplicate and two tissue samples placed in quadruplicate (272 array spots). Multiple tumors from nine dogs were present on the array and multiple samples from varying sites within the same tumor were present for twelve dogs. Additionally, non-melanoma stromal or control normal tissues were included. TMAs were hematoxylin and eosin-stained and evaluated via routine immunohistochemical procedures for melanoma cocktail (anti-melan A, anti-melanosome, and anti-tyrosinase), and antibodies to vimentin, MDM2 and p53. Samples scoring positive for MDM2 staining were then confirmed for positive staining with melanoma cocktail and re-evaluated for p53 staining. Positive staining was counted if at least one of the two duplicate samples could be evaluated for both MDM2 and melanoma cocktail on the TMA. Antibodies were purchased from Santa Cruz Biotechnology or Cell Marque. A total of 98 dogs and 189 spots/samples (132 tumors) met these criteria for evaluation for MDM2 protein expression by IHC. Of these 98 dogs, 18 dogs (17%) had melanocytic tumors positive for MDM2 staining in 33 spots/samples (25%). MDM2 staining was predominantly cytoplasmic highest intensity at junction between epithelial and subepithelial (submucosa, dermis). Staining was observed in both malignant pigmented and amelanotic melanoma and benign melanocytomas. Most intense staining (4+ cytoplasmic and nuclear) was observed in a benign cutaneous melanocytoma from a boxer that had additionally a malignant melanoma (negative for MDM2 staining on the array) and multiple cutaneous mast cell tumors.

### Nucleic acid extraction from tumor tissue and blood

Tissue was disrupted and homogenized in Buffer RLT plus (Qiagen AllPrep DNA/RNA Mini Kit), using the Bullet Blender, Next Advance, and transferred to a microcentrifuge tube containing Buffer RLT plus and 1.6 mm stainless steel beads or 0.9 mm–2.0 mm RNase free stainless steel beads. Blood leukocytes (buffy coat) were isolated from whole blood by centrifugation at room temperature and resuspended in Buffer RLT plus. All samples were homogenized, centrifuged at full speed, and lysates were transferred to Qiagen AllPrep spin columns. Genomic DNA and RNA were then purified following the manufacturer's protocol. DNA was quantified using the Nanodrop spectrophotometer and quality was accessed from 260/280 and 260/230 absorbance ratios. RNA was analyzed on the Agilent Bioanalyzer RNA 6000 Nano Chip to validate RNA integrity (RIN≥7.0).

### Library construction and next generation sequencing

Three μg of genomic DNA from each sample was fragmented to a target size of 300–350 base pairs (bp). Overhangs in the fragmented samples were repaired and adenine bases were ligated on. Diluted paired end Illumina adapters were then ligated onto the A-tailed products. Following ligation, samples were run on a 3% TAE gel to separate products. Ligation products at 300 bp and 350 bp were selected for each sample, isolated from gel punches, and purified. 2× Phusion High-Fidelity PCR Master Mix (Finnzymes; catalog#F-531L) was used to perform PCR to enrich for these products. Enriched PCR products were run on a 2% TAE gel and extracted. Products were quantified using Agilent's High Sensitivity DNA chip (catalog#5067–4626) on the Agilent 2100 Bioanalyzer (catalog#G2939AA).

Long insert whole genome libraries were constructed as previously described [126] with the following modifications: 1100ng inputs were used; following DNA fragmentation, a bead purification was performed at a 1:1.8 (sample volume: bead volume) ratio; a 1% size selection gel was used; and during library enrichment, 10 PCR cycles was used. Libraries were clustered onto Illumina V3 flowcells (San Diego, CA) using Illumina’s TruSeq PE Cluster Kit V3 (cat#PE-401-3001) and sequenced for paired 100bp reads using Illumina’s TruSeq SBS Kit V3 (cat#FC-401-3002, n = 3) on the Illumina HiSeq.

10 ng of total RNA was used to generate whole transcriptome libraries for RNA sequencing. Using the Nugen Ovation RNA-Seq System (cat#7100–08), total RNA was used to generate double stranded cDNA, which was amplified using Nugen's SPIA linear amplification process. Amplified cDNA was input into Illumina's TruSeq DNA Sample Preparation Kit–Set A (cat#FC-121-1001) for library preparation. In summary, 1 μg of amplified cDNA was fragmented to a target insert size of 300 bp and end repaired. Samples were then adenylated and indexed paired end adapters were ligated. Ligation products were run on a 2% TAE gel and size selected at 400 bp. Ligation products were isolated from gel punches and purified. Cleaned ligation products were input into PCR to enrich for libraries. PCR products were cleaned and quantified using the Agilent Bioanalyzer.

Tumor and normal libraries were prepared for paired end sequencing as described above. Clusters were generated using Illumina's cBot and HiSeq Paired End Cluster Generation Kits (catalog#PE-401-1001) and sequenced on Illumina's HiSeq 2000 using Illumina's HiSeq Sequencing Kit (catalog#FC-401-1001).

### Next generation sequencing data analysis

All informatic tools, versions, and flags are shown in S13 Table. BCL to FASTQ file conversion was performed using Illumina's BCL converter tool. Read alignment was performed with BWA (Burrows-Wheeler Aligner) v.0.7.8 [127] using the canine reference genome CanFam 3.1. Aligned BAMs were indel (insertion/deletion) realigned and recalibrated using GATK v3.3.0 [128, 129] and duplicate reads marked using Picard v1.128 (http://broadinstitute.github.io/picard/). Variants were called using Strelka v.1.0.13 [130], Seurat v2.6 [131] and MuTect v.1.1.4 [132] and calls were annotated according to dbSNP 151, SNPs on the Illumina CanineHD BeadChip, and SnpEff-3.5 [133]. Final somatic SNVs were called by at least 2/3 callers. LI-WGS data were utilized for CNV and SV detection. For CNV detection, read depths at every 100 bases across sequenced regions were determined. Next, normalized log_2_ fold-changes between tumor and normal were calculated and a smoothing window applied. Tumor allele frequencies of known heterozygous germline SNPs were utilized to evaluate potential false positives and correct biases. Finally, the Circular Binary Segmentation algorithm [134] was used to correct log_2_ fold-changes. For mutation burden metrics, a focal CNV is included if the log_2_ change is > = |2|. SV detection was performed utilizing Delly v0.76 [52]. A minimum tumor allele ratio of 0.10 and a minimum quality score of 20 is required for an SV to be called.

RNA sequencing data in FASTQ format was checked for quality using cycle-by-cycle quality plots and biases such as GC content. Reads were aligned to the canine reference genome CanFam 3.1 using STAR-2.4 to generate alignment files in BAM format [49]. Somatic SNVs were called with HaplotypeCaller (GATK v3.3.0) and verified in IGV. Transcript abundance in FPKMs (Fragments Per Kilobase of transcript per Million mapped reads) was obtained by CuffDiff v2.2.1 [134] and annotated using ENSEMBL (CanFam 3.1.68).

### Data access

Next generation sequencing data from this study have been submitted to the NCBI Biosample Database (http://www.ncbi.nlm.nih.gov/bioproject/389294) under project number PRJNA389294 with sample accession IDs SAMN07376261, SAMN07376262, SAMN07376263, SAMN07376264, SAMN07376265, SAMN07376266, SAMN07376267, SAMN07376268, SAMN07376269, SAMN07376270, SAMN07376271, SAMN07376272, and SAMN07376273.

### Pathway analysis

A list of 1,405 genes with single nucleotide variation or structural variation or copy number variation from the discovery cohort were analyzed using ClueGo4 [79], a Cytoscape plug-in, to create a functionally organized pathway network. Kappa scores were then used to measure association between the networks. Functional networks were created with a minimum Kappa score threshold of 0.5 and a minimum of 3 affected genes in every network forming at least 10% of the total associated genes in that particular network. The genes were assigned to the networks based on the predefined pathways from KEGG, REACTOME and Wiki Pathways. 97 pathways were obtained, all with Benjamini-Hochberg corrected p-value <0.05. These pathways were grouped together based on inter-term kappa score and named by the most significant pathway in the respective groups.

### PCR amplification and Sanger sequencing analysis

PCR amplification of 15 genes (*NRAS*, *KRAS*, *BRAF*, *GNA11*, *GNAQ*, *PTPRJ*, *TP53*, *MDM2*, *BAP1*, *CDK4*, *PTEN*, *c-KIT*, *MITF* and *NF1*) was performed using primers targeting all coding exons (S4 Table). All amplification reactions were performed using Platinum Taq DNA Polymerase #10966–034 (Life Technologies; Carlsbad, CA). Briefly, each primer pair was mixed with 10 ng of genomic DNA and subjected to the following cycling parameters: 94°C for 2 min., 3 cycles at each temperature: 30 sec. at 94°C, 30 sec. at 60–57°C, 45 sec. at 72°C; 25 cycles: 30 sec. at 94°C, 30 sec. at 62°C, 45 sec. at 72°C; final extension of 5 min. at 72°C. PCR amplicons were sequenced using M13 forward and reverse primers at the Arizona State University’s DNA Lab (Tempe, AZ).

### Array comparative genomic hybridization

Oligo array CGH (aCGH) was performed by co-hybridization of tumor (test) DNA and a common reference DNA sample, where the latter comprised an equimolar pool of genomic DNA samples from multiple healthy individuals of various breeds. DNA was labeled using an Agilent SureTag Labeling Kit (Agilent Technologies, Santa Clara, CA) with all test samples labeled with Cyanine-3-dCTP and the common reference sample labeled with Cyanine-5-dCTP. Fluorochrome incorporation and final probe concentrations were determined using routine spectrophotometric parameters with readings taken from a Nanodrop1000. Fluorescently labeled test and reference samples were co-hybridized to Canine G3 180,000 feature CGH arrays (Agilent, AMADID 025522) for 40 h at 65°C and 20 rpm, as described previously [135, 136]. Arrays were scanned at 3 μm using a high-resolution microarray scanner (Agilent,Model G2505C) and data extracted using Feature Extraction (v10.9) software. Scan data were assessed for quality by the ‘Quality Metrics’ report in Agilent’s Feature extraction software (v10.5) (Agilent Technologies).

### SNP array genotyping

SNP genotyping was performed using the Illumina CanineHD array (cat#WG-440-1001). Per manufacturer’s protocol, 200ng of DNA was first denatured then neutralized with 0.1N NaOH before amplification at 37°C for 24 hours. The amplified DNA was then enzymatically fragmented and precipitated using 100% 2-propanol before drying for one hour at room temperature. After resuspension the fragmented DNA was then denatured and loaded onto the CanineHD BeadChip and hybridized for 16 hours at 48°C. BeadChips were washed, a single base extension of hybridized primers added followed by multi-layer staining of the primers. Arrays were then washed, coated with the XC4 reagent (Illumina) and dried under vacuum for one hour. Coated arrays were read on the HiScan system and data visualized using the Illumina GenomeStudio Genotyping 2.0 software with an average sample call rate of 97%.

### aCGH and SNP array data analysis

For both aCGH and SNP arrays, copy number data were analyzed with NEXUS Copy Number v8.0 software (Biodiscovery Inc., CA, USA). For cross-platform comparisons, LI-WGS BAMs were also analyzed utilizing Nexus software. CNVs were identified using a FASST2 segmentation algorithm with a significance threshold of 5.5×10^−6.^ Aberrations were defined as a minimum of three consecutive probes with log2 tumor: reference value of >1.14 (high gain), 1.13 to 0.2 (gain), −0.23 to −1.1 (loss), <−1.1 (big loss). Recurrent CNVs within each subtype were determined within NEXUS using an involvement threshold of 50%. Significance of these regions was then determined in NEXUS using the GISTIC algorithm (to identify regions with a statistically high frequency of CNVs over background) with a G-score cut off of G>1.0 and a significance of Q<0.05. CNV frequency comparisons amongst sample groups were performed in NEXUS using Fisher’s exact test with differential threshold of >50% and significance p<0.05. Significance of each probe between the two groups was calculated in NEXUS using a Mann–Whitney test for median comparison.

## Supporting information

S1 Fig Histopathological features of canine malignant melanoma. Hematoxylin and eosin staining for three subtypes of canine melanoma included in this study. 100x magnification on the left and 400x magnification on the right. (A) Canine mucosal melanoma. (B) Canine acral melanoma. (C) Canine cutaneous melanoma.
(TIF)

S2 Fig Extended trinucleotide mutation spectrum. (A) The distribution of somatic single nucleotide mutation types in the discovery cohort as total SNVs. (B) Dinucleotide context of C>T transitions (dipyrimidines versus non-dipyrimidines) in the discovery cohort. (C) Mutational signatures based on trinucleotide context and frequency of somatic single nucleotide mutations in the discovery cohort.
(TIF)

S3 Fig CIRCOS plots displaying the genomic landscape of 7 canine melanomas. The outer ring comprises the chromosomal karyotype with SNVs shown on the adjacent internal track as blue triangles. CNVs are displayed in the inner ring showing gains in red and losses in green. Rearrangements are displayed as lines connecting two loci.
(TIF)

S4 Fig CNV concordance plots between the three platforms in the discovery cohort. (TIF)

S5 Fig Alignment of human and canine KIT. The query represents the human protein with accession number NP_000213.1. This is compared to the subject canine protein ENSCAFP00000039467 which shares an 88% identity over 100% of the protein length.
(TIF)

S6 Fig Alignment of human and canine PTPRJ. The query represents the human protein with accession number NP_002834.3. This is compared to the subject canine protein ENSCAFP00000012172 which shares a 73% identity over 97% of the protein length. The red box indicates the highly conserved protein tyrosine phosphatase catalytic domain.
(TIF)

S7 Fig RNAseq fragments per kilobase of transcript per million mapped reads (FPKMs). (TIF)

S8 Fig MDM2 staining of canine melanoma. (A) Representative samples from a canine melanoma TMA stained with MDM2 showing increased expression in two samples. (B) 100x magnification of cytoplasmic MDM2 staining with highest intensity at junctions between epithelial and subepithelial layers (see arrows).
(TIF)

S9 Fig Alignment of human and canine TP53. The query represents the human protein with accession number NP_000537.3. This is compared to the subject canine protein ENSCAFP00000024579 which shares a 81% identity over 100% of the protein length.
(TIF)

S10 Fig Frequency of PTPRJ mutations across human cancers. (A) The spectrum of PTPRJ alterations within samples available through cBioPortal. (B) The distribution of all reported PTPRJ sequence mutations in cBioPortal.
(TIF)

S1 Table Canine melanoma sample information. ND = no data; WGS = whole genome sequencing; LI = long insert; mRNA-seq = mRNA sequencing; SNP-A = single nucleotide polymorphism array.
(XLSX)

S2 Table Whole genome sequencing metrics. * The average number of times a base is read or spanned by mate paired reads. Calculated using the formula C = N(2L+I)/G where C is the physical coverage, N = number of aligned reads, L = read length (the 2 multiplier denotes paired end sequencing), G = size of canine genome and I = inter-read base pair distance for PE seqencing. **The proportion of the genome that could be genotyped accurately at a minimum read depth of 20 at a single locus; PF = Passing Filter.
(XLSX)

S3 Table Somatic coding mutations identified in canine melanoma discovery cohort*. *Sequencing approaches include next generation sequencing (short-insert whole-genome (SI-WGS), long-insert whole genome (LI-WGS), or mRNA-Seq) and Sanger sequencing. **Sanger validation uninformative n/a = <4 reads detected at this locus; ND = no data.
(XLSX)

S4 Table Somatic nsSNVs indentified by targeted Sanger sequencing in canine melanoma. *Predicted Deleterious by PROVEAN (http://provean.jcvi.org/index.php). **Associated germline unavailable; ^+^Annotated using RefSeq- no ENSEMBL available; ^1^Genomic position based on CanFam2 build.
(XLSX)

S5 Table Somatic copy number alterations identified in canine melanoma discovery cohort. (XLSX)

S6 Table Copy number variations identified by SNP array and GISTIC in canine melanoma. (XLSX)

S7 Table Copy number variations identified by SNP Array in canine melanoma by sample. (XLSX)

S8 Table Somatic structural variants identified in canine melanoma discovery cohort. (XLSX)

S9 Table LOH identified by SNP array in canine melanoma by sample. (XLSX)

S10 Table MDM2 amplifications and immunohistochemistry. (XLSX)

S11 Table Significantly dysregulated pathways in canine melanoma. (XLSX)

S12 Table PTPRJ mutations in human cancers. (XLS)

S13 Table Informatic tools, versions and flags. (XLSX)
