# Supplementary figures and images for: Somatic inactivating PTPRJ mutations and dysregulated pathways identified in canine malignant melanoma by integrated comparative genomic analysis
Source: PLoS Genet. 2018 Sep 6;14(9):e1007589. doi: 10.1371/journal.pgen.1007589 (PMC6126841; doi:10.1371/journal.pgen.1007589)

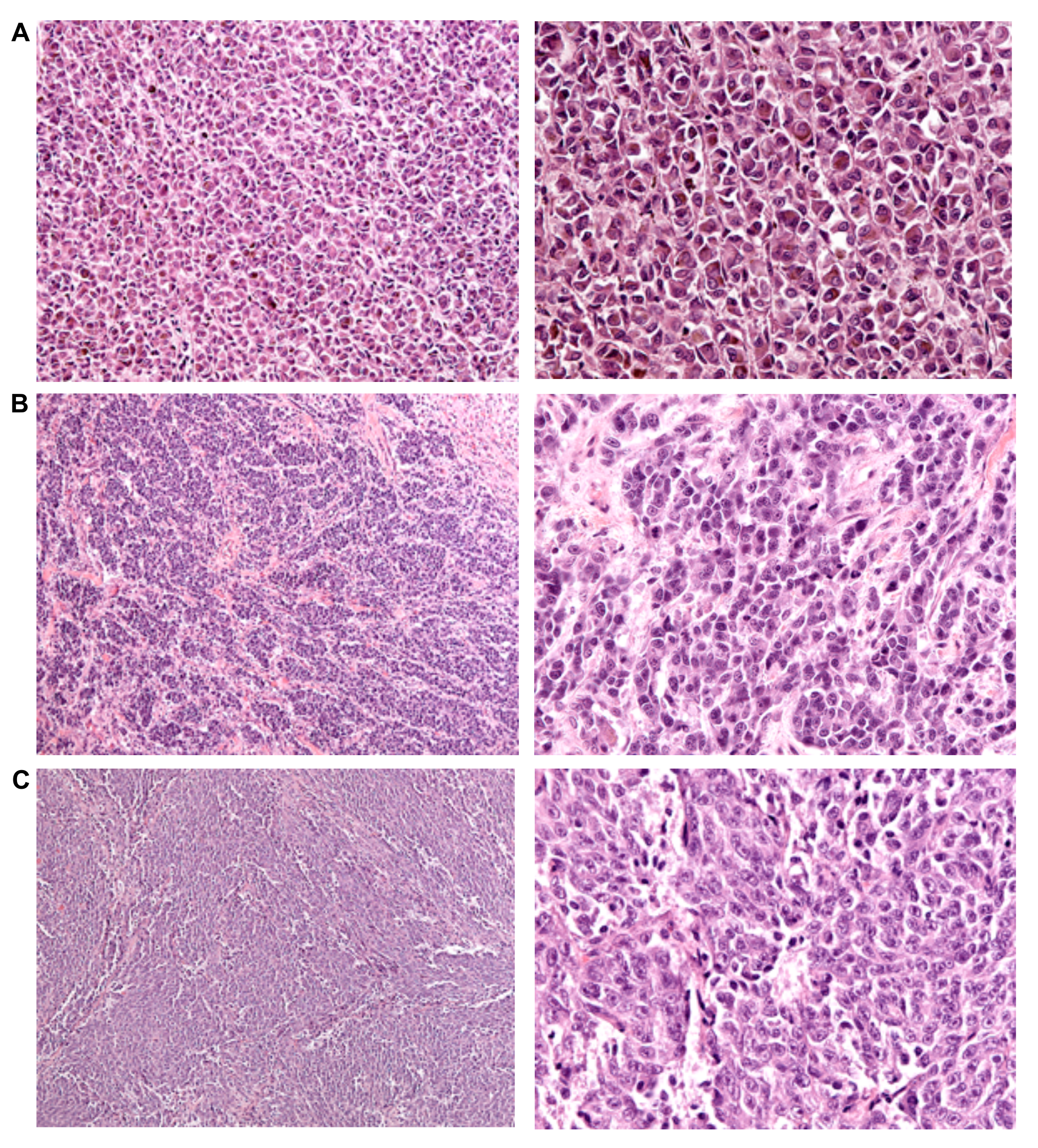

Supplement: S1 Fig — Hematoxylin and eosin staining for three subtypes of canine melanoma included in this study. 100x magnification on the left and 400x magnification on the right. (A) Canine mucosal melanoma. (B) Canine acral melanoma. (C) Canine cutaneous melanoma. (TIF) [file pgen.1007589.s001.tif]

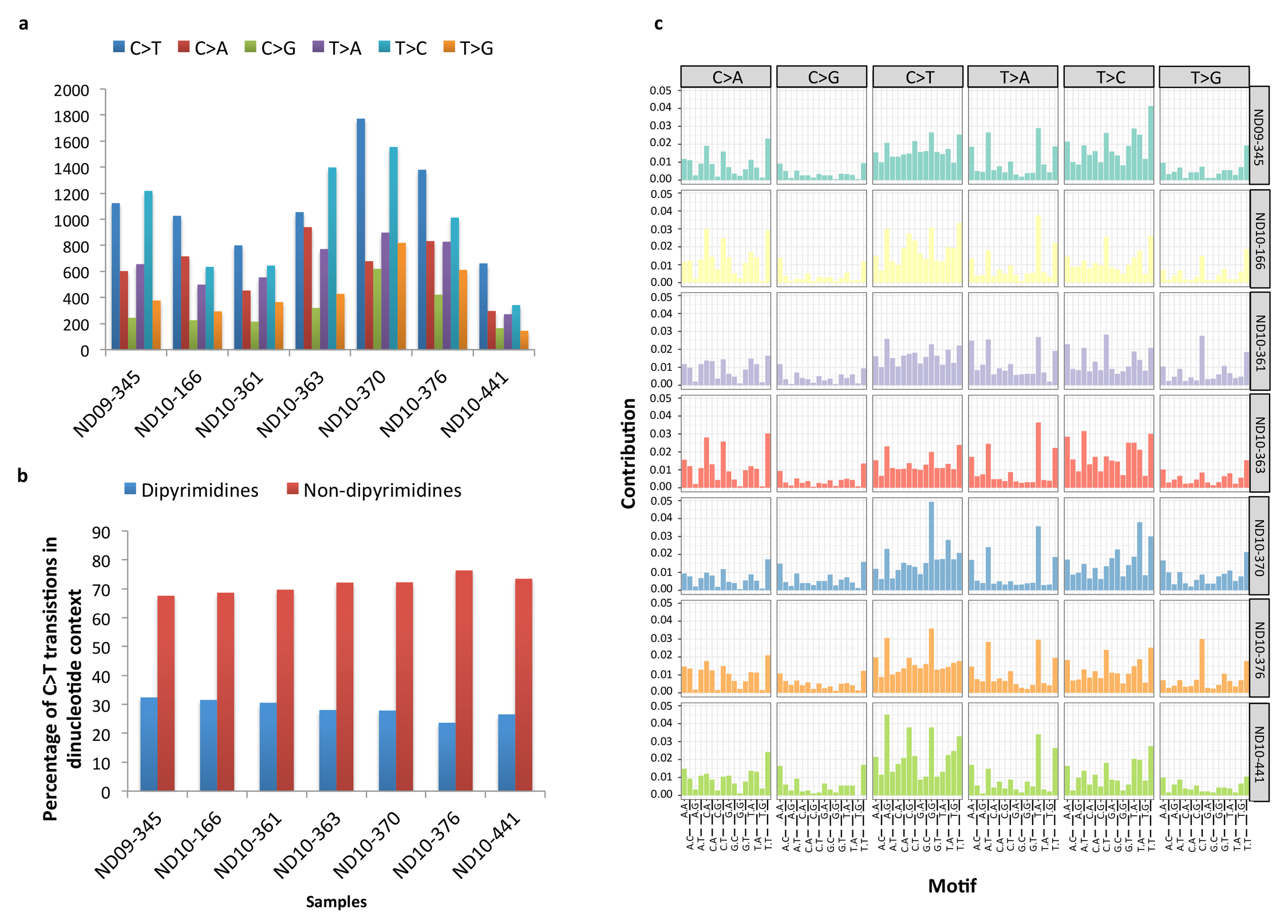

Supplement: S2 Fig — (A) The distribution of somatic single nucleotide mutation types in the discovery cohort as total SNVs. (B) Dinucleotide context of C>T transitions (dipyrimidines versus non-dipyrimidines) in the discovery cohort. (C) Mutational signatures based on trinucleotide context and frequency of somatic single nucleotide mutations in the discovery cohort. (TIF) [file pgen.1007589.s002.tif]

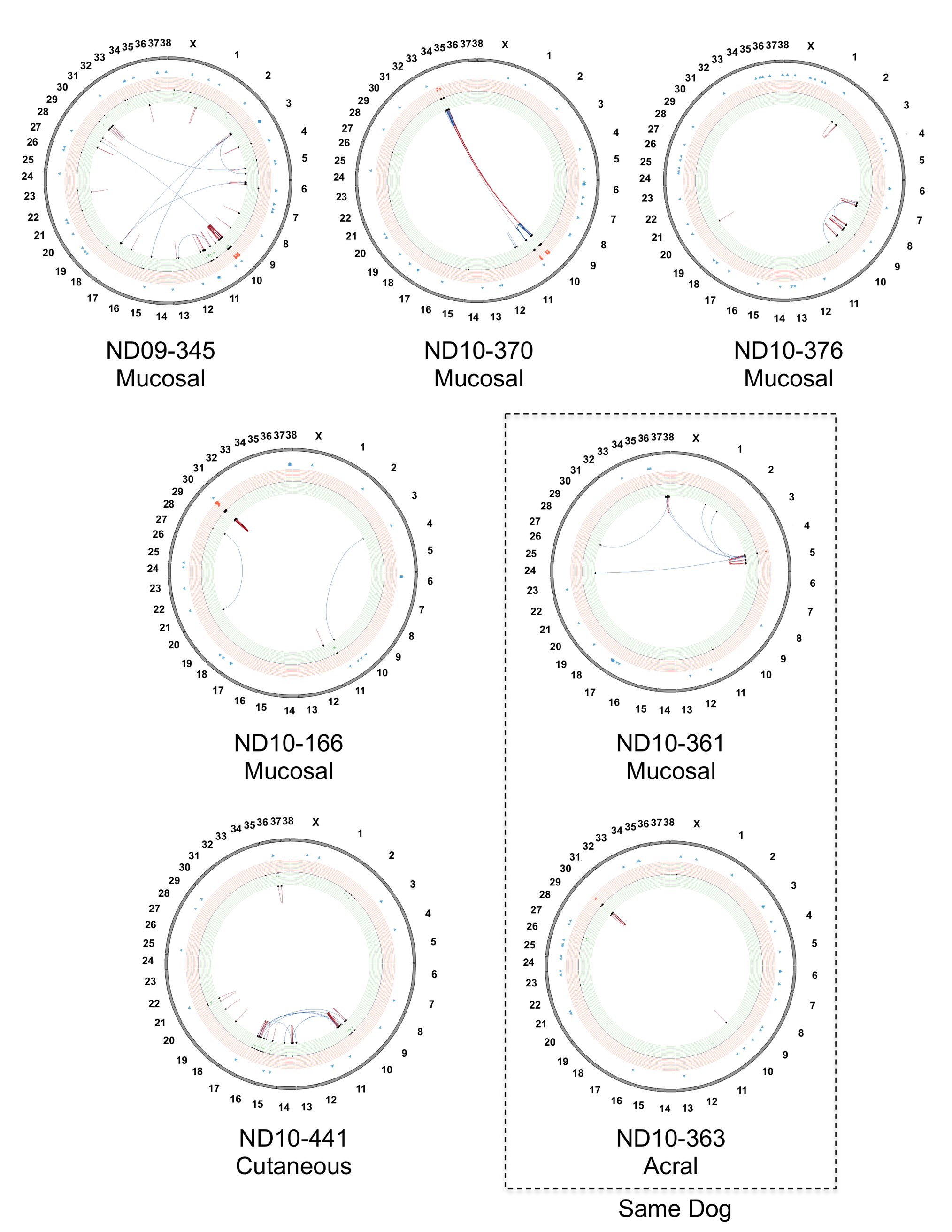

Supplement: S3 Fig — The outer ring comprises the chromosomal karyotype with SNVs shown on the adjacent internal track as blue triangles. CNVs are displayed in the inner ring showing gains in red and losses in green. Rearrangements are displayed as lines connecting two loci. (TIF) [file pgen.1007589.s003.tif]

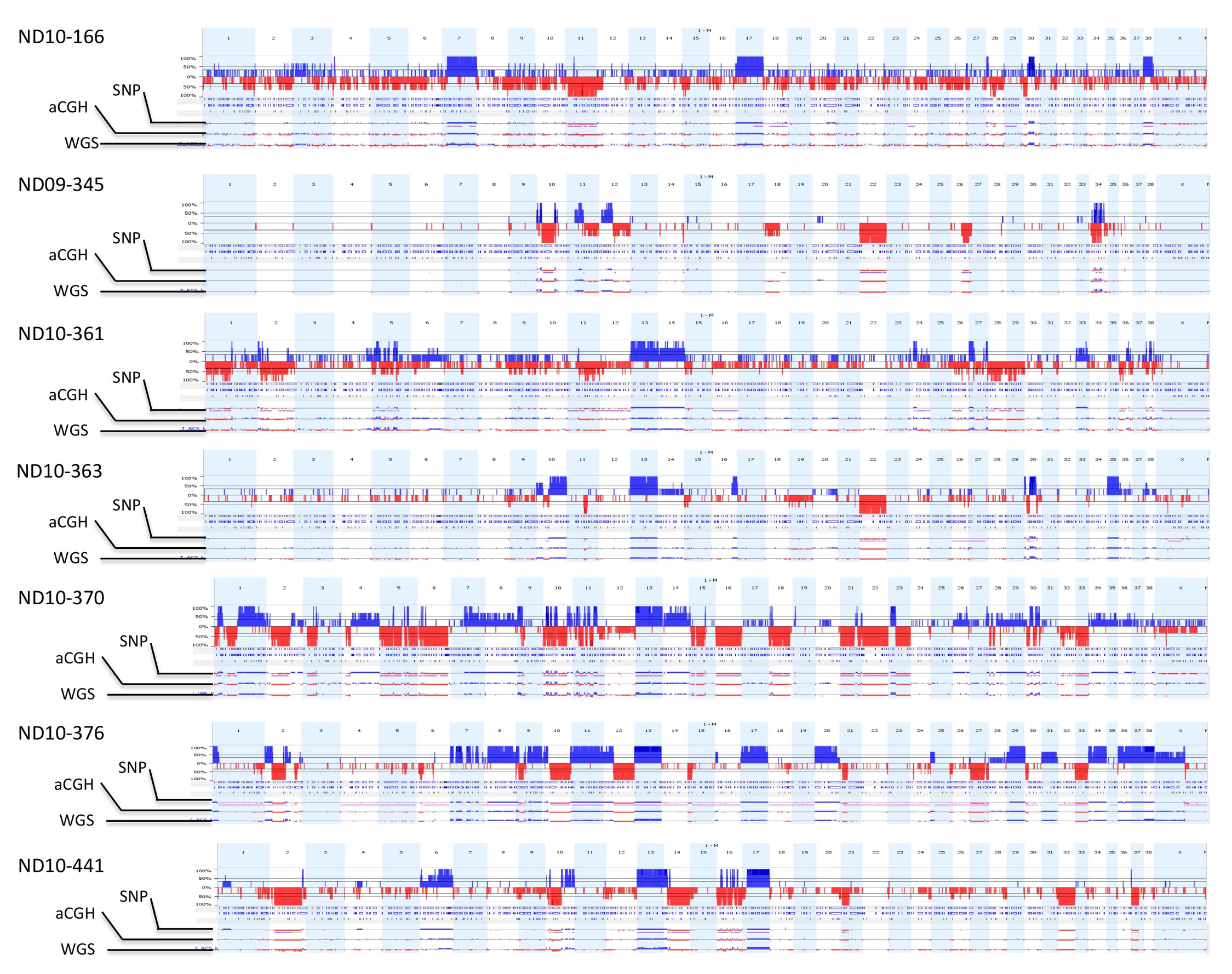

Supplement: S4 Fig — (TIF) [file pgen.1007589.s004.tif]

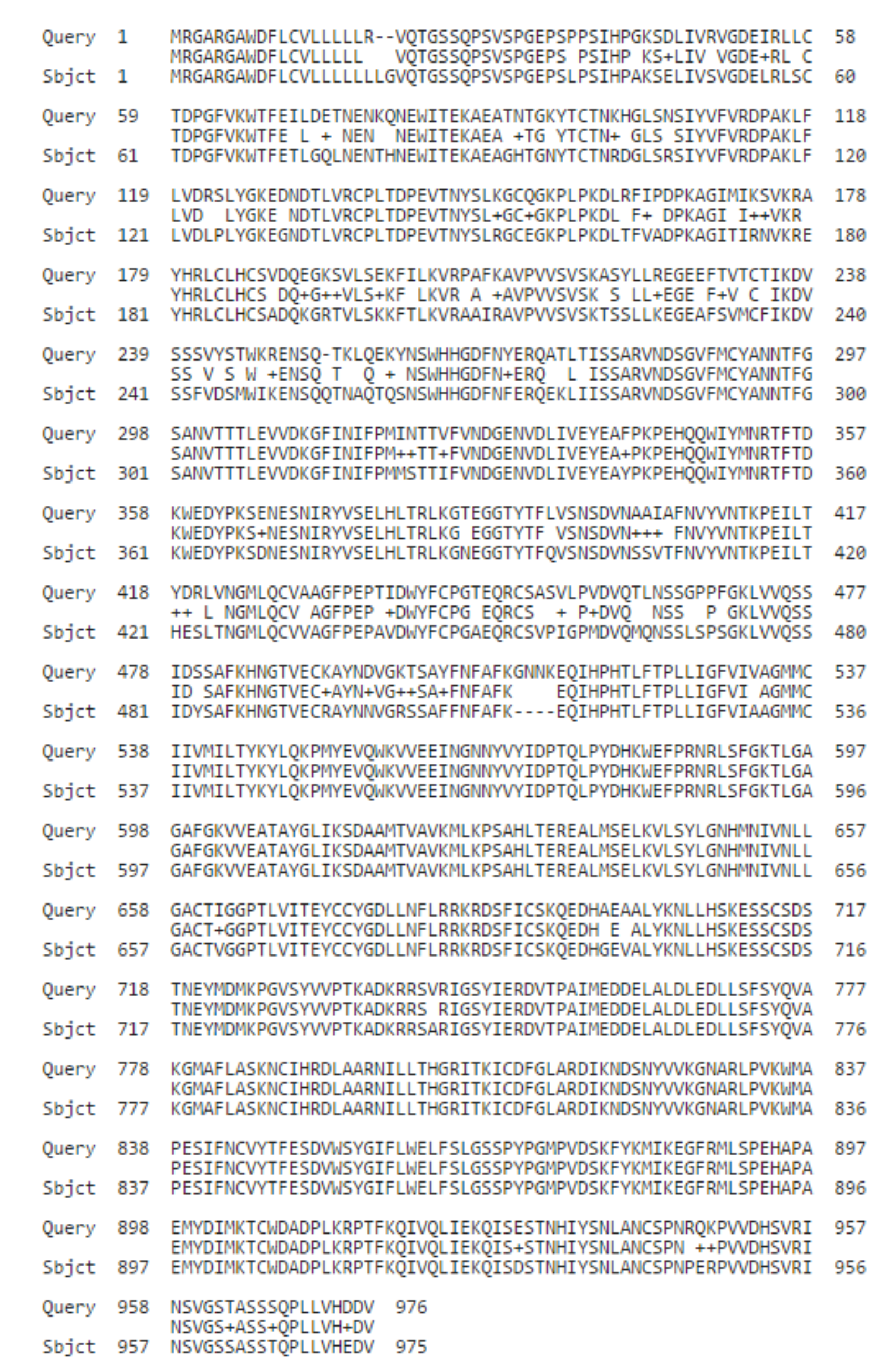

Supplement: S5 Fig — The query represents the human protein with accession number NP_000213.1. This is compared to the subject canine protein ENSCAFP00000039467 which shares an 88% identity over 100% of the protein length. (TIF) [file pgen.1007589.s005.tif]

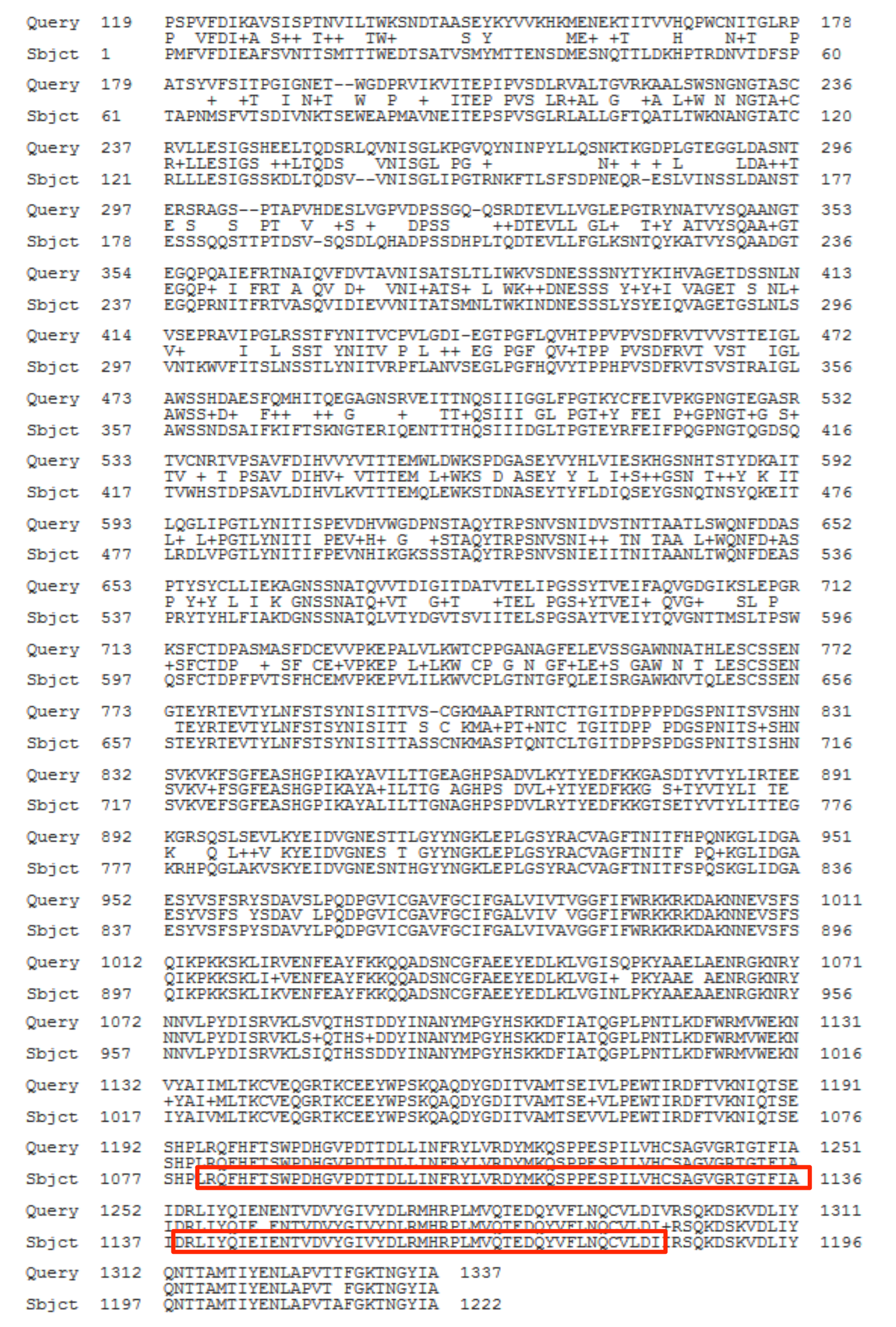

Supplement: S6 Fig — The query represents the human protein with accession number NP_002834.3. This is compared to the subject canine protein ENSCAFP00000012172 which shares a 73% identity over 97% of the protein length. The red box indicates the highly conserved protein tyrosine phosphatase catalytic domain. (TIF) [file pgen.1007589.s006.tif]

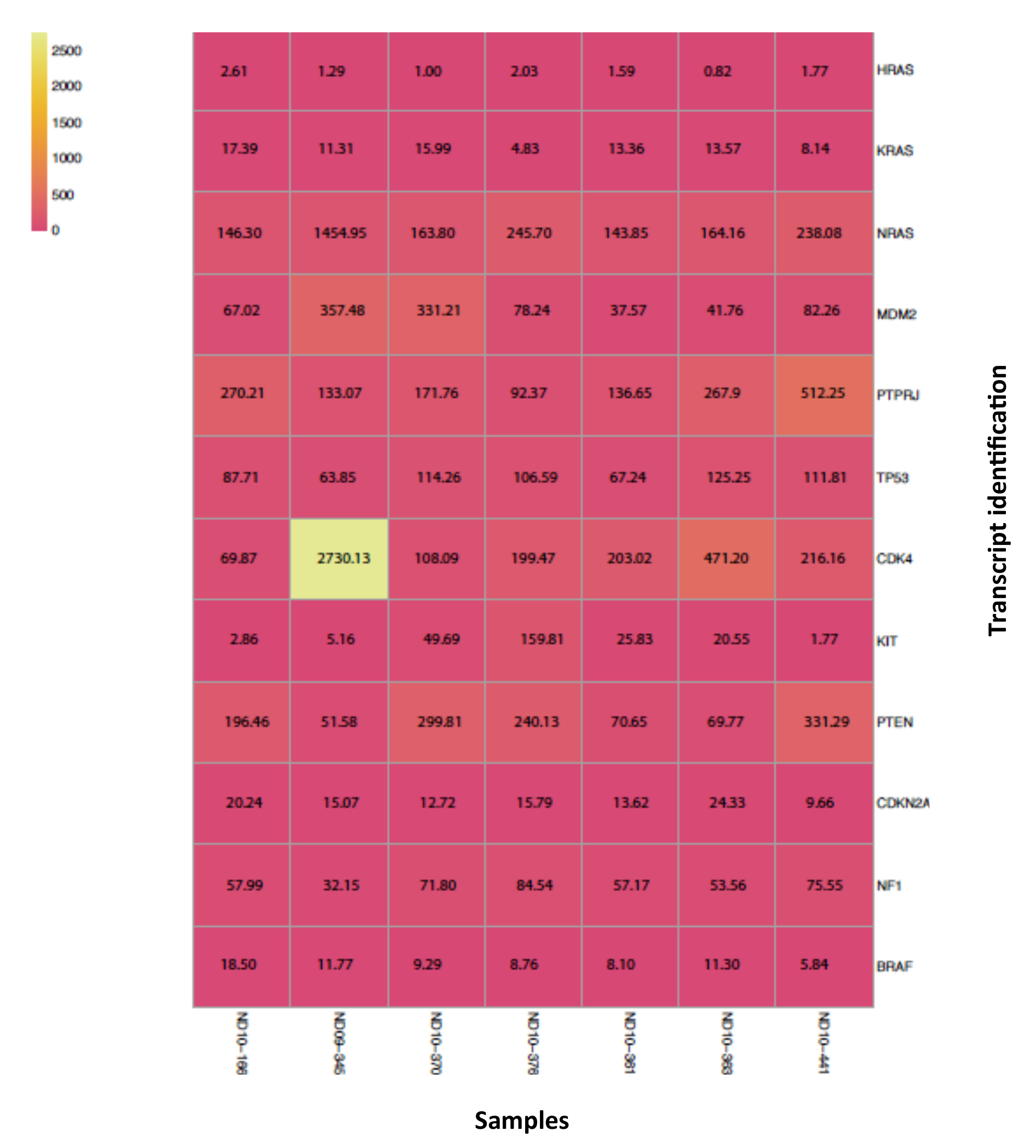

Supplement: S7 Fig — (TIF) [file pgen.1007589.s007.tif]

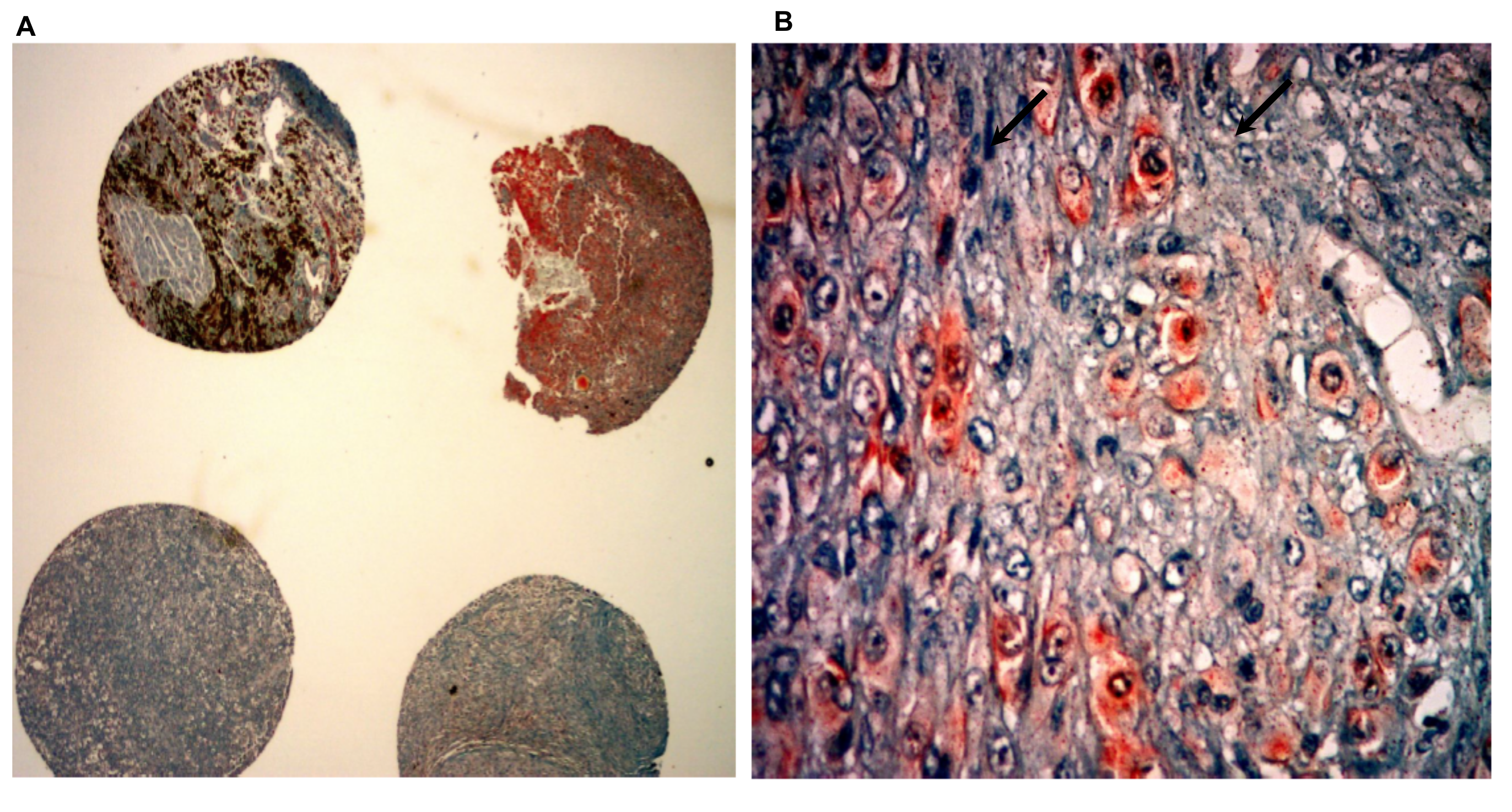

Supplement: S8 Fig — (A) Representative samples from a canine melanoma TMA stained with MDM2 showing increased expression in two samples. (B) 100x magnification of cytoplasmic MDM2 staining with highest intensity at junctions between epithelial and subepithelial layers (see arrows). (TIF) [file pgen.1007589.s008.tif]

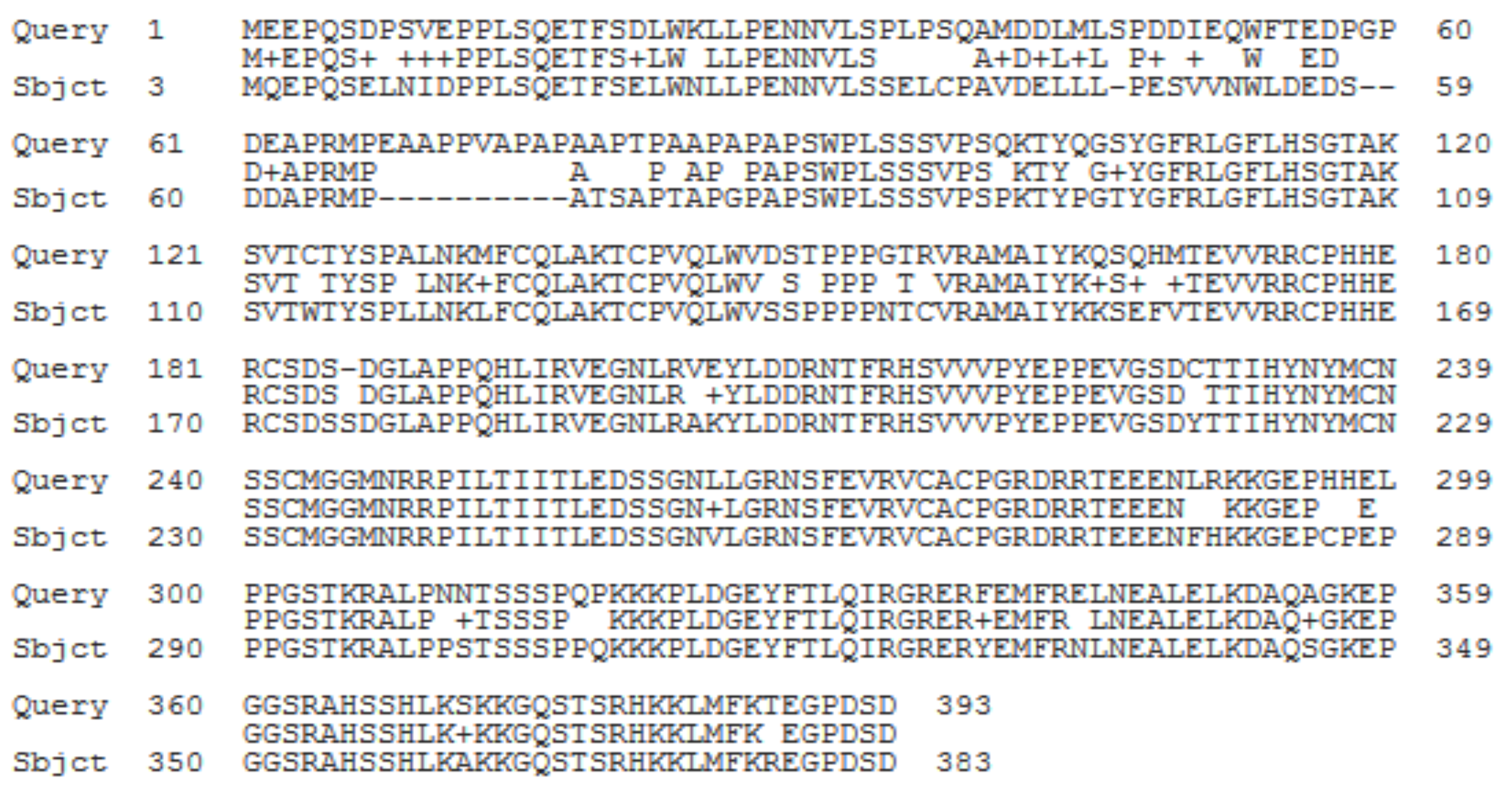

Supplement: S9 Fig — The query represents the human protein with accession number NP_000537.3. This is compared to the subject canine protein ENSCAFP00000024579 which shares a 81% identity over 100% of the protein length. (TIF) [file pgen.1007589.s009.tif]

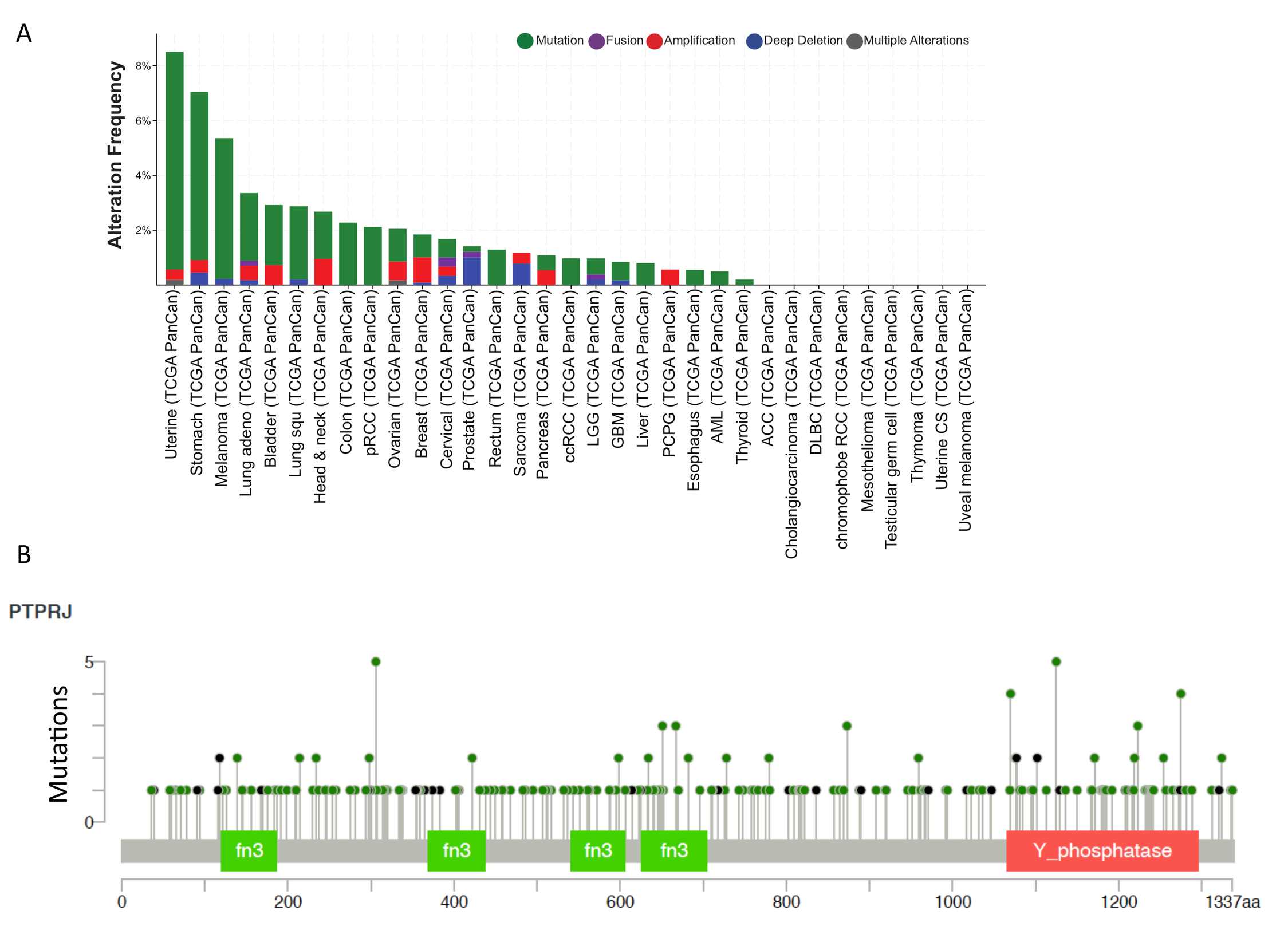

Supplement: S10 Fig — (A) The spectrum of PTPRJ alterations within samples available through cBioPortal. (B) The distribution of all reported PTPRJ sequence mutations in cBioPortal. (TIF) [file pgen.1007589.s010.tif]
